# Dissecting Alzheimer disease in Down syndrome using mouse models

**DOI:** 10.3389/fnbeh.2015.00268

**Published:** 2015-10-13

**Authors:** Xun Yu Choong, Justin L. Tosh, Laura J. Pulford, Elizabeth M. C. Fisher

**Affiliations:** ^1^Department of Neurodegenerative Disease, Institute of Neurology, University College LondonLondon, UK; ^2^The LonDownS ConsortiumLondon, UK

**Keywords:** Alzheimer disease, APP, Down syndrome, mouse models, trisomy 21

## Abstract

Down syndrome (DS) is a common genetic condition caused by the presence of three copies of chromosome 21 (trisomy 21). This greatly increases the risk of Alzheimer disease (AD), but although virtually all people with DS have AD neuropathology by 40 years of age, not all develop dementia. To dissect the genetic contribution of trisomy 21 to DS phenotypes including those relevant to AD, a range of DS mouse models has been generated which are trisomic for chromosome segments syntenic to human chromosome 21. Here, we consider key characteristics of human AD in DS (AD-DS), and our current state of knowledge on related phenotypes in AD and DS mouse models. We go on to review important features needed in future models of AD-DS, to understand this type of dementia and so highlight pathogenic mechanisms relevant to all populations at risk of AD.

## Introduction: AD-DS, the most common genetic form of AD

Down syndrome (DS) is a complex, heterogeneous disorder caused by the presence of an extra copy of human chromosome 21. Trisomy 21 is a common condition, with an incidence of 1 in 750 live births (Parker et al., 2010). Prevalence in many countries is growing due to increasing maternal age, the greatest risk factor for DS (Loane et al., 2013), together with rises in DS life expectancy (Yang et al., 2002; Bittles and Glasson, 2004). In Northern Europe, for example, the number of people aged over 40 years with DS is approximately double what it was in 1990, and in the UK this age group accounts for a third of the estimated 40,000 people with DS (Wu and Morris, 2013).

The clinical presentation of DS varies extensively and includes features present in all individuals, such as cognitive deficits, and those seen in only some people, such as heart defects (Zigman, 2013; Jensen and Bulova, 2014). Alzheimer disease (AD) pathology is found in the brains of virtually all people with DS by 40 years of age (Wisniewski et al., 1985; Mann and Esiri, 1989), and trisomy 21 causes an increased risk of dementia such that approximately one third of the DS population has AD (“AD-DS”) by the age of 60, with an estimated lifetime prevalence of 90% for all people with DS (Prasher and Krishnan, 1993; Holland et al., 1998; Coppus et al., 2006; Margallo-Lana et al., 2007; McCarron et al., 2014). However, while AD-DS is one of the largest contributors to morbidity and mortality in DS (Coppus et al., 2008), not all individuals develop dementia, even by 70 years of age (Krinsky-McHale et al., 2008; Ghezzo et al., 2014). Thus, the DS population has the most common genetic form of early-onset AD, caused by trisomy 21. Studying AD-DS allows investigation of the initial pathogenic events leading to AD and the development of dementia, relevant to both people with DS and to the general population.

One approach to dissecting human disease is through studying mouse models, and a large number of transgenic strains have been generated to understand specific aspects of AD pathology, most of which have human gene mutations that give rise to rare early-onset familial Alzheimer disease (FAD; Braidy et al., 2012; Webster et al., 2014). In the last decade, chromosome engineering techniques have enabled the generation of an array of DS mouse models that will allow us to dissect the genetic contribution of chromosome 21 (Hsa21), or regions of the mouse genome syntenic to Hsa21, to DS phenotypes. These models recapitulate a wide range of DS features, including neurobiological, behavioral and aging-related aspects (Zhang et al., 2012b; Ruparelia et al., 2013). Thus, in the study of AD-DS, mouse models of DS offer an increasingly important approach to understanding pathogenic mechanisms, so informing us about pathways and networks relevant to all populations at risk of dementia.

Here, we present an overview of clinical features of AD-DS, compared to other genetic forms of AD, to highlight human phenotypes that may be assessed in mechanistic studies of mouse models. We then give examples of data from DS mouse models compared to transgenic mice modeling aspects of AD pathology, to illustrate informative findings from both types of model. We also offer examples of potentially helpful data for investigating AD-DS from the outcomes of overexpressing single genes from Hsa21. Finally, we consider the important features for mouse models to enhance our understanding of AD-DS, and therefore the pathogenetic mechanisms relevant to all AD. For brevity, citations may not necessarily be the original papers, but useful reviews or later references.

## Genetic forms of AD, including AD-DS

The *APP* gene lies on Hsa21 and encodes the amyloid precursor protein that is at the heart of the amyloid cascade hypothesis of Alzheimer disease (Glenner and Wong, 1984; Hardy and Higgins, 1992; Hardy and Selkoe, 2002). This hypothesis was generated partly from the observation that extracellular plaques in brains of people with AD are composed of Aβ peptides that are products of APP metabolism. The hypothesis suggests that abnormal APP metabolism initiates AD pathogenesis by triggering a set of events that result in Aβ aggregation, particularly of the Aβ42 peptide, in these extracellular plaques. This leads to the formation of intracellular neurofibrillary tangles, primarily composed of the protein tau, and eventually loss of synapses and neurons. The relationship between the histopathological features of AD and dementia is not yet clear (Castellani and Perry, 2014).

The amyloid cascade hypothesis is currently the most widely-accepted paradigm guiding investigations of AD pathogenesis, and is supported at least in part by the rare cases of FAD caused by different mutations in *APP*, and in the presenilin genes *PSEN1* and *PSEN2* that affect APP processing. *APP* mutations may, for example, result in an increase in total Aβ production, or a relative increase in Aβ species associated with pathogenicity (Ryan and Rossor, 2010).

Importantly for understanding AD-DS, the link between *APP* and AD also extends to gene dose: in rare forms of FAD, duplication of the wildtype *APP* locus alone (“Dup-APP”) is sufficient to cause highly penetrant early-onset AD (Rovelet-Lecrux et al., 2006; Sleegers et al., 2006). Dup-APP cases demonstrate that the three doses of *APP* arising from trisomy 21 are likely to be causative for AD-DS. Conversely, although very rare, partial trisomy 21 excluding *APP* (i.e., with two “doses” of *APP*) does not appear to lead to AD (Prasher et al., 1998; Korbel et al., 2009).

While people with DS and Dup-APP are at high risk of dementia, presumably in both cases because of *APP* triplication, there are some intriguing differences in their AD-related clinical features (Wiseman et al., 2015). Examining the effects of different *APP* genotypes may therefore provide insights into the modulation of *APP* pathogenesis. Table 1 shows key examples of phenotypes in AD-DS and how these compare with Dup-APP, FAD due to other *APP* mutations (primarily point mutations) and late-onset sporadic AD (SAD). Mutations in *PSEN1* and *PSEN2*, which do not map to Hsa21, are not included.

**Table 1:** **Comparison of phenotypes from different genetic forms of human Alzheimer disease**.

**Phenotype**		**AD-DS: three copies of wildtype *APP***	**FAD (Dup-APP): three copies of wildtype *APP***	**FAD (*APP* mutations): Usually heterozygous for a mutant *APP* allele. *N.B. these mutations do not necessarily act by the same mechanisms***	**SAD: two copies of wildtype *APP***
**CLINICAL SYMPTOMS**					
Cognition	Incidence and age of onset of dementia	Less than 40 years of age, < 5% people with DS have dementia but prevalence doubles every 5 years; by 55–60 years, 50–70% of DS have AD (Tyrrell et al., 2001; Hartley et al., 2015)	Dementia onset ~42–59 years of age (Cabrejo et al., 2006)	Dementia onset ~45–60 years of age (Ryan and Rossor, 2010)	Dementia onset usually >65 years of age (Querfurth and LaFerla, 2010)
		Total prevalence across lifespan estimated at ~90% (McCarron et al., 2014)			
	Pre-clinical cognitive symptoms	Pre-existing cognitive impairments complicate diagnosis of AD in DS (Zigman, 2013)	No apparent pre-symptomatic cognitive impairment (Cabrejo et al., 2006; Rovelet-Lecrux et al., 2006)	Pre-symptomatic impairment of verbal memory and IQ; early progressive impairment of episodic memory (Rovelet-Lecrux et al., 2006; Hooli et al., 2012)	Mild cognitive impairment (cognitive symptoms, notably memory problems, which do not significantly affect function) precedes dementia (Albert et al., 2011), although only 5–20% go on to develop dementia
		Memory deficits may occur up to 3 years before dementia diagnosis (Krinsky-McHale et al., 2002)			
	Clinical presentation of dementia	Amnestic presentation similar to AD after taking into account pre-existing baseline intellectual deficits	Slow and progressive memory impairment and loss of cognition (Sleegers et al., 2006)	Most cases have similar amnestic presentation to SAD (Pilotto et al., 2013)	Progressive deficits in episodic memory, semantic knowledge, working memory, and attention (Weintraub et al., 2012)
		However, changes in behavior and personality are more common than SAD (Krinsky-McHale et al., 2000; Devenny et al., 2002; Ball et al., 2008)			
	Sex differences	No difference between sexes (Coppus et al., 2006)	Not reported	Not reported	Women at higher risk (Musicco, 2009)
Epilepsy		Up to 84% AD-DS experience seizures (Mendez and Lim, 2003; De Simone et al., 2010)	Up to 57% exhibit seizures (Rovelet-Lecrux et al., 2006)	Seizures described in at least four different APP mutations (Kumar-Singh et al., 2000; Murrell et al., 2000; Grabowski et al., 2001; Pasalar et al., 2002)	Up to 10–20% of patients exhibit seizures (Mendez and Lim, 2003; Palop, 2009)
**CLASSICAL AD NEUROPATHOLOGY: Aβ** **AND TAU**					
Aβ accumulation and deposition	Intracellular Aβ	Intraneuronal accumulation of Aβ42 has been seen at 3 years of age. Levels decline as diffuse and dense core plaques develop (Mori et al., 2002)	Intraneuronal accumulation of Aβ40 in post mortem brain. No intraneuronal Aβ42 detected (Cabrejo et al., 2006)	Not reported	Intracellular staining found in post mortem SAD tissue (LaFerla et al., 2007)
	Extracellular Aβ	Earliest extracellular deposition found at 8 years of age (Leverenz and Raskind, 1998) Aβ40 undetectable in plaques in DS brain < 50 years of age. Proportion of Aβ40 in plaques gradually increases until =50 years of age 42% of dense-core plaques comprise of Aβ40 (Iwatsubo et al., 1995) Amyloid plaques universal in DS people by age 31 (Leverenz and Raskind, 1998; Hartley et al., 2015)	Parenchymal lesions predominantly composed of Aβ42. Vascular amyloid predominantly Aβ40 (Cabrejo et al., 2006; Rovelet-Lecrux et al., 2006) Abundant parenchymal and vascular lesions as both dense-core and diffuse plaques (Cabrejo et al., 2006; Guyant-Marechal et al., 2008)	Increased Aβ42/Aβ40 ratio and/or increased Aβ production (Tanzi, 2012). Rare APP A673T mutant confers protection against AD pathology (Peacock et al., 1993; Hashimoto and Matsuoka, 2014) Pattern and progression of amyloid plaque deposition is largely identical to SAD. However, mutations within the Aβ sequence can cause increased deposition in the vasculature (Pilotto et al., 2013)	Accumulation of Aβ42 and Aβ40 into amyloid plaques. Aβ42 is more abundant in plaques (Serrano-Pozo et al., 2011) Amyloid plaque deposition progresses in a stereotypical fashion characterized by Thal phases I-V (Thal et al., 2002) Highest accumulation of plaques found in layers II–IV of the isocortex (Braak and Braak, 1991; Serrano-Pozo et al., 2011)
	Cerebral Amyloid Angiopathy (CAA) and Intra-cranial Hemorrhage (ICH)	CAA pathology common in DS. ICH is rare (Mann, 1988a; McCarron et al., 1998; Naito et al., 2008)	CAA is ubiquitous (Cabrejo et al., 2006; Sleegers et al., 2006; Rovelet-Lecrux et al., 2007; Kasuga et al., 2009) ICH in 20–50% of cases (Cabrejo et al., 2006; Rovelet-Lecrux et al., 2007; Guyant-Marechal et al., 2008; Kasuga et al., 2009)	CAA is in a large number of FAD mutations but not all (Ryan and Rossor, 2010) Arctic and Dutch APP mutations both affect residue 693 but only patients with Dutch mutation develop CAA and ICH (Basun et al., 2008; Ryan and Rossor, 2010)	~50–80% of cases have CAA, deposits primarily composed of Aβ40 (Jellinger et al., 2007; Serrano-Pozo et al., 2011) ICH in ~3% of SAD cases, possibly related to hypertension (Jellinger et al., 2007)
Neurofibrillary tangles		NFTs present in almost all people with DS by age 45. Density of NFTs triples between age 40–50 (Wisniewski et al., 1985; Goedert et al., 1992) NFT density correlates more strongly with clinical dementia rating than Aβ plaque count (Margallo-Lana et al., 2007) NFTs only manifest subsequent to dense-core amyloid plaques (Hartley et al., 2015)	NFTs consistent with late stage AD present at time of death (Rovelet-Lecrux et al., 2006)	Different FAD mutations exert highly variable effects on NFTs, from absence of NFTs in Arctic mutations to severe pathology (Ryan and Rossor, 2010)	Stereotypical spatiotemporal progression of NFTs begins in the allocortex of the medial temporal lobe with six stages of development, distinguished by Braak stages (Braak and Braak, 1991) Increased levels of total tau and phospho-tau correlate with increase in SAD severity (Wallin et al., 2006; Serrano-Pozo et al., 2011)
Neuronal loss and brain atrophy		Neuronal atrophy follows SAD pattern but trend for less relative cell loss and atrophy compared to SAD (Mann, 1988b) Selective loss of BFCNs from as early as 5.5 months of age. Progressive loss of neurons in the Nucleus basalis of Meynert during aging (Casanova et al., 1985; McGeer et al., 1985)	Diffuse cortical atrophy with parietal dominance and neuronal loss (Cabrejo et al., 2006; Sleegers et al., 2006; Rovelet-Lecrux et al., 2007; Guyant-Marechal et al., 2008; Kasuga et al., 2009)	Similar neuronal atrophy pattern to SAD with a slightly more severe medial-temporal pattern (Pilotto et al., 2013)	Characteristic loss of neurons and white matter (Querfurth and LaFerla, 2010). Neuronal loss correlates with NFTs (Gómez-Isla et al., 1997; Serrano-Pozo et al., 2011) Basal forebrain atrophy correlates with Aβ burden (Kerbler et al., 2015)
**OTHER FEATURES OF AD PATHOLOGY**					
Synaptic loss and dysfunction		Synaptic protein expression decreased in aging DS brain (Downes et al., 2008) GABA levels decreased in post-mortem hippocampus and temporal cortex (Reynolds and Warner, 1988; Seidl et al., 2001; Martínez-Cué et al., 2014)	Not reported	Not reported	Synapse loss is best correlate of cognitive decline and precedes neuronal loss (Ingelsson et al., 2004; Scheff et al., 2007) GABA significantly reduced in post mortem cortical but not subcortical brain regions. *In vivo* evidence of GABA loss in parietal cortex (Seidl et al., 2001; Bai et al., 2014)
Oxidative stress and proteostasis		Some proteins oxidatively modified differently in DS and control groups, suggesting DS subjects vulnerable to oxidative damage (Di Domenico et al., 2014)	Not reported	Not reported	Increased levels of oxidative stress are a hallmark of SAD pathology and linked to aging (Madeo, 2013)
Endosomal dysfunction		Endosome enlargement, alterations in morphology and function in young DS (pre-AD) and DS fibroblasts (Jiang et al., 2010)	Not reported	Enlarged endosomes modulated by ApoE status (Cataldo et al., 2001)	Enlarged endosomes detected in preclinical stages (Cataldo et al., 1997, 2000) Aβ accumulates within late endosomes in AD brain (Takahashi et al., 2002)
Neuroinflammation		Dystrophic microglia and absence of activated microglia at 40 years of age, coincident with tau pathology (Xue and Streit, 2011) Increased astrocytic activation in early DS, increases with age and correlates with amyloid deposition (Royston et al., 1999)	Not reported	Not reported	Hyper-reactive, dystrophic microglia associated with dense-core plaques and NFTs (McGeer et al., 1987; Streit et al., 2009) Reactive astrocytes locate early to dense-core plaques, triggered by Aβ (Itagaki et al., 1989; Pike et al., 1995) Higher neuroinflammation in younger (<80) compared to older patients with SAD, suggesting importance in early stages of disease (Hoozemans et al., 2011)

*Down syndrome (AD-DS), familial AD due to APP duplications (Dup-APP), familial AD due to APP mutations (FAD), and sporadic Alzheimer disease (SAD) Abbreviations: BFCNs, basal forebrain cholinergic neurons; CAA, cerebral amyloid angiopathy; GABA, γ-Aminobutyric acid; ICH, intra-cranial hemorrhage; ID, intellectual disability; NFT, neurofibrillary tangles*.

However, a difficulty in analysing phenotypes is the considerable heterogeneity in clinical presentation within each *APP* genotype, even within families with the same mutation. For example, there is a wide variety of non-cognitive symptoms and behavioral changes across all four AD genotypes, including personality changes (Nelson et al., 2001; Ball et al., 2008), hallucinations (Sleegers et al., 2006; Basun et al., 2008; Guyant-Marechal et al., 2008), paranoia (Sleegers et al., 2006; Pilotto et al., 2013), and delusions (Burns et al., 1990), some of which are associated with cognitive decline (Adams and Oliver, 2010). Another important issue in diagnosing AD in AD-DS is that dementia is an additional cognitive deficit acquired on top of the baseline cognitive impairment found in people with DS: distinguishing between cognitive deficits due to intellectual disability, and decline at early stages of AD, is therefore an important challenge. However, diagnosis of dementia by experienced clinicians has been shown to be accurate in DS, and even more reliable than recent operational dementia criteria (Sheehan et al., 2015). Further, a few clinical features stand out in AD-DS—a striking example, albeit one of unknown relevance to AD, is seizure susceptibility in adulthood, which appears heightened by *APP* duplication, as both AD-DS (84%) and Dup-APP (57%) have significantly higher rates of seizures than SAD (10–20%). This may indicate specific pathways that are progressively disrupted by *APP* duplication, resulting in damaging electrical activity in the brain.

Dup-APP and FAD caused by *APP* mutations are relatively rare, and much information about these conditions remains to be gathered, for example, on synaptic dysfunction, oxidative stress and neuroinflammation. In contrast, AD-DS arises in a population with a well-defined genetic basis and a sizeable prevalence, which means it is of great value for investigating AD pathogenesis for everyone at risk of dementia.

## Modeling DS, including AD-DS, in mice

**Figure 1:**
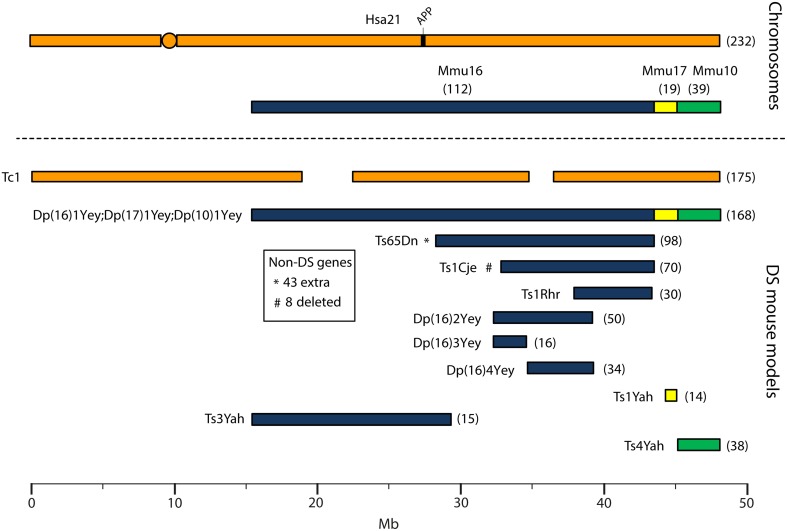
**Human chromosome 21 (Hsa21), orthologous mouse chromosomes (Mmu), and key mouse models of Down syndrome**. Diagram representing Hsa21 and its alignment with syntenic regions on Mmus 16, 17, and 10. The orange circle represents the human centromere and mouse models are color-coded and aligned according to the chromosomal segment for which they are trisomic. Numbers in brackets represent the number of protein-coding Hsa21 orthologous genes within each region or mouse model, according to Ensembl release 79 and the breakpoints published in papers referenced here. The Tc1 mouse is the only model which carries Hsa21, though genomic rearrangements and deletions (indicated by breaks in the chromosome) mean the mouse is functionally trisomic for only ~75% of Hsa21 genes (Gribble et al., 2013). All other mouse models carry duplications of mouse orthologues. The Dp1(16)Yey;Dp1(17)Yey;Dp1(10)Yey (or Ts1Yey;Ts3Yey;Ts2Yey) mouse was generated by crossing together three partial trisomy models (Yu et al., 2010a) and spans the entirety of the Hsa21-syntenic regions. The Ts65Dn mouse (Davisson et al., 1993) contains a freely segregating segment of Mmu16, however it is also trisomic for 43 extra protein-coding genes on the centromeric section of Mmu17 that are not relevant to DS (indicated by an asterisk (^*^) and accompanying text box; Duchon et al., 2011; Reinholdt et al., 2011). The Ts1Cje mouse (Sago et al., 1998) also contains a monosomy of eight protein-coding genes on Mmu12, irrelevant to the DS phenotype (indicated by “#” and accompanying text box. Gene numbers are based on Ensembl release 79, compared to the original seven monosomic genes detailed in Duchon et al., 2011). Other mice are Ts1Rhr or Dp1(16)Rhr mice (Olson et al., 2004); Ts1Yah mice (Pereira et al., 2009); Ts3Yah (previously published as Ts2Yah; Brault et al., 2015); and Ts4Yah mice (previously published as Ts3Yah mice; Herault et al., 2009). Other useful examples of mouse models include the Ts43H model (not shown) which is partially trisomic for Mmu17 including some genes with ortholog on Hsa21 (Vacík et al., 2005). The scale is in megabase pairs (Mb).

Human chromosome 21 has synteny with the mouse genome, such that its ortholog genes are found in three blocks with conserved order and gene orientation on mouse chromosomes 10 (Mmu10), Mmu16, and Mmu17 (Hattori et al., 2000; Dierssen et al., 2009); the mouse *App* gene lies on Mmu16 (Figure 1). Mice with precisely-defined trisomies (or monosomies) have been generated, now usually by chromosome engineering (Brault et al., 2006; Tybulewicz and Fisher, 2006), to provide a set of models that are segmentally trisomic for regions orthologous to Hsa21 (Davisson et al., 1993; Sago et al., 1998; Olson et al., 2004; Li et al., 2007; Herault et al., 2009; Pereira et al., 2009; Yu et al., 2010a; Liu et al., 2011, 2014; Brault et al., 2015).

Generating many models with different partial trisomies creates a mapping panel in which individual phenotypes may be assessed in several strains, and so assigned to specific trisomic chromosomal region(s). As all DS phenotypes presumably arise from abnormal gene dosage, candidate genes that when present in three copies give rise to all or part of the phenotype, can be chosen from the trisomic critical region. Individual candidate genes can then be studied, for example, in overexpression or knockout models, to assess the effects of different copy numbers of the gene. Figure 1 is an overview of DS mouse models and the chromosomal segments for which they are trisomic. Table 2 details the gene content for each DS mouse model shown, including protein-coding and non-protein-coding genes relevant to human trisomy 21.

**Table 2:** **Trisomic region and triplicated gene content in Down syndrome mouse models shown in Figure 1 compared with Hsa21 (Ensembl release 79)**.

		**Protein-coding genes**		**Non-protein-coding genes**		**Total genes**		**% Protein-coding genes from Hsa21**
	**Hsa21**	**232**		**648**		**880**		
**DS mouse model**	Official MGI name^*^	**Mouse genes**	**Hsa21 genes**	**Mouse genes**	**Hsa21 genes**	**Mouse genes**	**Hsa21 genes**	
Tc1	B6;129S-Tc(Hsa21)1TybEmcf/J	–	175	–	Undetermined	–	N/A	75
Dp(16)1Yey	B6.129S7-Dp(16Lipi-Zbtb21)1Yey/J	149	112	112	6	261	118	48
Dp(17)1Yey	B6;129S7-Dp(17Abcg1-Rrp1b)3Yey/J	19	18	6	0	25	18	8
Dp(10)1Yey	B6;129S7-Dp(10Prmt2-Pdxk)2Yey/J	55	39	20	1	75	40	17
Ts65Dn^**^	B6EiC3Sn a/A-Ts(1716)65Dn	133	98	71	3	204	101	42
Ts1Cje^***^	B6.Cg-T(12;16)1Cje/CjeDnJ	76	70	51	1	127	71	30
Ts1Rhr	B6.129S6-Dp(16Cbr1-Fam3b)1Rhr/J	32	30	20	0	52	30	13
Dp(16)2Yey	129-Dp(16Tiam1-Kcnj6)6Yey/J	53	50	37	1	90	51	22
Dp(16)3Yey	129-Dp(16Tiam1-Il10rb)8Yey/J	18	16	12	0	30	16	7
Dp(16)4Yey	129-Dp(16Ifnar1-Kcnj6)10Yey/J	35	34	24	1	59	35	15
Ts1Yah	B6;129P2-Dp(17Abcg1-Cbs)1Yah/Orl	15	14	4	0	19	14	6
Ts3Yah (previously Ts2Yah)	B6;129P2-Dp(16Hspa13-App)2Yah/Orl	19	15	45	5	64	20	6
Ts4Yah (previously Ts3Yah)	B6.Cg-Dp(10Prmt2-Cstb)3Yah/Orl	54	38	20	1	74	39	16
**TRISOMIC/MONOSOMIC REGIONS AND GENE CONTENT IRRELEVANT TO Hsa21 AND ITS SYNTENIC REGIONS IN MICE**								
Ts65Dn^**^	B6EiC3Sn a/A-Ts(1716)65Dn	43	–	36	–	79	–	
Ts1Cje^***^	B6.Cg-T(12;16)1Cje/CjeDnJ	8	–	4	–	12	–	

* *Mouse genome informatics site that includes the official mouse strain names www.informatics.jax.org; the shaded line shows number of Hsa21 genes*.

** indicates gene content of Ts65Dn and

*** *indicates gene content of Ts1Cje mice*.

The most complete mouse model to date, *Dp(10)1Yey/*+*;Dp(16)1Yey/*+*;Dp(17)1Yey/*+, is trisomic for all Hsa21 syntenic regions and was generated by crossing three DS mouse models, each carrying duplications of the respective Hsa21 orthologous regions on Mmu10, Mmu16 and Mmu17 (Li et al., 2007; Yu et al., 2010a,b; Figure 1). However, the vast majority of studies relating to AD-DS have been performed on the Ts65Dn mouse, as this has been an extremely important “standard model” of DS for many years, prior to the development of newer strains by chromosome engineering (Davisson et al., 1993; Reeves et al., 1995; Table 2). The Ts65Dn mouse carries a Robertsonian translocation resulting in trisomy of ~42% of the protein-coding genes orthologous to Hsa21, but it also has 79 additional genes (including long non-coding sequences) from Mmu17 that are outside the Hsa21 region of synteny, and these need to be taken into account when analysing phenotypes (Duchon et al., 2011; Reinholdt et al., 2011). These extra triplicated genes that do not relate to DS happen to include non-Hsa21 genes, such as *SYNJ2* and *TIAM2* that have Hsa21/Mmu16 paralogues (*SYNJ1, TIAM1*), which may complicate phenotype-genotype correlations (Duchon et al., 2011). Other triplicated genes in Ts65Dn irrelevant to DS include several genes encoding dynein light chains that may influence endosomal trafficking, and so potentially affect neuronal phenotypes (Hartley et al., 2015).

A different type of mouse model of DS is the “humanized” transchromosomic “Tc1” mouse that carries a freely-segregating Hsa21 (O'Doherty et al., 2005), which is functionally trisomic for ~75% of Hsa21 protein-coding genes (Gribble et al., 2013). However, this extra chromosome is rearranged, and lost stochastically at different rates in different mouse tissues—thus, Tc1 mice are mosaic for the human chromosome. With respect to AD research, the *APP* gene is not functionally trisomic in Tc1 mice because of a rearrangement that has occurred by chance, so this animal expresses just the two endogenous copies of mouse *App* (Sheppard et al., 2012).

While many DS mouse models have been published, there is no single complete model, and the usefulness of these strains lies in their comparative and complementary use in studying genotype-phenotype relationships, including AD-related phenotypes (Table 3). These studies enable us to map critical dosage-sensitive genes because each locus is likely expressed at trisomic levels, mimicking human DS transcription. We can also study the interactions of Hsa21 dosage-sensitive genes with the rest of the genome (Hsa21 and non-Hsa21), as well as effects exerted by aneuploidy *per se*.

**Table 3:** **Examples of AD phenotypes studied in DS mouse models, and related findings in *APP* transgenic strains described in Table 4**.

**Phenotype**	**DS models**		***APP* transgenic models**
Cognitive deficits	Learning and memory deficits widely demonstrated, mostly in young mice (Das and Reeves, 2011) Differentiating between early cognitive impairment and neurodegeneration in old age is a challenge (Ruparelia et al., 2013). One study suggests learning deficits in Ts65Dn worsen with age, but due to lack of motivation or motor impairment rather than neurodegeneration (Sanders et al., 2009)		Working memory, episodic memory, executive function, and attention deficits in *APP* transgenic mice from young ages (3–5 months; Webster et al., 2014) Memory impairments linked to neurotoxicity as a result of Aβ oligomers (Lesné et al., 2006) or insoluble Aβ deposits (Westerman et al., 2002) Behavioral deficits deteriorate with age in some *APP* transgenic mice (Hsiao et al., 1996; Van Dam et al., 2003)
Long-term potentiation (LTP)	Hippocampal LTP deficits reported in all models trisomic for Mmu16 regions syntenic to Hsa21, apart from Ts2Yah for which no LTP data is available (Das and Reeves, 2011) LTP increased in Dp1(17)Yey and unaltered in Dp1(10)Yey (Yu et al., 2010b) LTP deficits observed in Tc1 suggest compromised entorhinal cortex input into the dentate gyrus, contributing to impaired CA3 and CA1 function (Witton et al., 2015)		LTP studies have produced often contradictory measurements within the same mouse models (Pozueta et al., 2013) Aberrant neuronal activity is a prominent feature; restoring inhibitory synaptic activity may rescue network hypersynchrony, memory deficits and early mortality (Sanchez et al., 2011; Verret et al., 2012; Stargardt et al., 2015)
Aβ accumulation and deposition	APP protein and mRNA expression	In Ts65Dn, APP protein increases to trisomic levels from 6 months in the striatum (Hunter et al., 2003), and from 10 months in cortex and hippocampus (Seo and Isacson, 2005; Contestabile et al., 2006) APP mRNA in Ts65Dn remains similar to disomic levels at 5 months but increases at 12 months (Choi et al., 2009)	*APP* transgenic mice generally overexpress human *APP* with FAD mutations at levels at least 5x endogenous mouse *App*. *APP* transgene transcription is directed by artificial promoters (Table 4) allowing expression in the central nervous system, usually from embryonic or early postnatal age (Crews et al., 2010; Balducci and Forloni, 2011; Hall and Roberson, 2012)
	APP metabolism	In Ts65Dn, total APP CTF levels increased in hippocampus, enriched in synaptosomes and early endosomes from 6 months (Salehi et al., 2006; Lockrow et al., 2009) In Ts65Dn no difference in Aβ42/40 ratios, low levels of larger (~115 kDa) SDS-stable Aβ oligomers (Salehi et al., 2006; Choi et al., 2009; Peng et al., 2009)	In line with the overexpression of *APP*, Aβ levels are generally overexpressed, with some models expressing FAD mutations driving an increase in Aβ42/40 ratios (Crews et al., 2010)
Tau	Neurofibrillary pathology	In aged Ts65Dn mice increased tau and reelin detected in granules in hippocampus and olfactory bulb (Kern et al., 2011) No tau neurofibrillary tangles detectable in Tc1 and Ts1Cje brains (O'Doherty et al., 2005; Shukkur et al., 2006; Sheppard et al., 2012)	*APP* transgenic mice fail to produce neurofibrillary tangles without additional mutations introduced in presenilin or tau (Kokjohn and Roher, 2009)
	Tau hyper-phosphorylation	In Ts65Dn, Ts1Cje and Tc1, increased tau phosphorylation in hippocampus and cortex at various phosphorylation sites (Shukkur et al., 2006; Liu et al., 2008; Sheppard et al., 2012) In Tc1 this was detected in old (20 months) but not young (2 months) mice (Sheppard et al., 2012) Unphosphorylated tau decreased in Ts1Cje mice (Shukkur et al., 2006)	Hyperphosphorylation of tau and its regulation have primarily been studied in *APP* transgenic mice with additional mutations in presenilin and/or tau Hyperphosphorylated tau is detectable in some *APP* transgenic mouse models (Kokjohn and Roher, 2009; Crews et al., 2010)
	Regulation of tau phosphorylation	Increased phosphorylation of GSK-3β in Tc1 and Ts1Cje (Shukkur et al., 2006; Sheppard et al., 2012). Increased phosphorylation of AKT in Tc1 and Ts65Dn (Siarey et al., 2006; Sheppard et al., 2012) CDK5 expression upregulated in Ts65Dn but not in Tc1 (Pollonini et al., 2008; Sheppard et al., 2012). No difference in CDK5 activators p25/p35 levels detected in both Ts1Cje and Tc1 (Shukkur et al., 2006; Sheppard et al., 2012)	
Neuronal loss and dysfunction	Loss and dysfunction of Basal Forebrain Cholinergic Neurons (BFCNs)	Reduced BFCN numbers and cell size in Ts65Dn mice from 12 months (Cooper et al., 2001; Salehi et al., 2006) ChAT activity increased in 10-month but no different from control in 19-month Ts65Dn (Contestabile et al., 2006) Distribution of cholinergic neurons in dentate gyrus altered in Ts65Dn (Cooper et al., 2001; Salehi et al., 2006) All above alterations not observed in Ts1Cje and Ts65Dn:App^+∕+∕−^ mice, both of which are disomic for *App* (Salehi et al., 2006)	Loss of BFCNs observed in APP23 and APP(V717I; Choi et al., 2013). No loss of BFCNs observed in APP23 (Boncristiano et al., 2002) and Tg2576 (Apelt et al., 2002) Decreased ChAT and AChE activity in basal forebrain nuclei of APP23 (Van Dam et al., 2005)
	Loss and dysfunction of noradrenergic neurons	Degenerative morphology and loss of noradrenergic neurons in rostral LC in Ts65Dn at 12 months but not 4 months (Lockrow et al., 2011b; Fortress et al., 2015)	Noradrenaline levels declined with aging in TgCRND8 hippocampus (Francis et al., 2012). No overt cell loss in LC in old APP23 and PDAPP mice, although neurons decreased in size in PDAPP (Szot et al., 2009; Francis et al., 2012)
**OTHER FEATURES POTENTIALLY RELEVANT TO AD**			
Epilepsy	5–10x increased rates of audiogenic seizures and seizure-related death in 21-day old Ts65Dn mice, attenuated by mGluR5 antagonists (Westmark et al., 2010)		Epileptiform activity and spontaneous non-convulsive seizures frequently observed in *APP* transgenic mice, from young ages (Born, 2015). Whether this is caused by overproduction of Aβ (Palop, 2009) or is an artifact of *APP* overexpression during development (Born et al., 2014) is unclear
Synaptic loss and dysfunction	Synaptic and dendritic abnormalities	In Ts65Dn, increased average synapse size with no change in synaptic number or density (Hernández-González et al., 2015) In Ts65Dn, dendritic spines are enlarged, less dense, and redistributed on principal neurons; arborizations are poorly developed. Similar but less severe observations in Ts1Cje (Dierssen et al., 2003; Belichenko et al., 2004) In Tc1, reduced synaptic size, complexity and density observed in hippocampus (Witton et al., 2015); decreased dendritic mushroom spines (associated with memory) at 3 months and increase in stubby spines (Haas et al., 2013) Ts1Rhr fewer thin spines (associated with learning) at 3 weeks of age (Haas et al., 2013)	Loss and alterations in dendritic spines and synapses are early features of neuronal pathology in *APP* transgenic mice models, before onset of plaque deposition and cognitive deficits. Synaptic deficits correlate well with soluble Aβ (Pozueta et al., 2013) Reduced density of mushroom-type spines of CA1 hippocampal region in two *APP* transgenic mouse models (Perez-Cruz et al., 2011)
Oxidative stress and proteostasis	Oxidative stress markers increased in young and old Ts65Dn mice (Lockrow et al., 2009; Shichiri et al., 2011; Di Domenico et al., 2015) Impaired mitochondrial function and increased ROS production in Ts1Cje cortical astrocyte and hippocampal neuronal cultures (Shukkur et al., 2006)		Oxidative stress increased and precedes Aβ deposition in *APP* transgenic mice. Increased Aβ levels lead to mitochondrial impairments (Eckert et al., 2010; Ye et al., 2012; Meraz-Ríos et al., 2014)
		
Endosomal dysfunction	Enlarged EEs in BFCNs and expression of EE proteins detected from 6 months in Ts65Dn, increasing in number with age (Cataldo et al., 2003; Salehi et al., 2006) EEs not enlarged in Ts1Cje and Ts65Dn-App^+∕+∕−^ mice, both of which are disomic for *App* (Cataldo et al., 2003) Axonal transport disruption selectively impaired for endosomal cargo in Ts65Dn mice (Salehi et al., 2006)		No enlargement of EEs observed in APP22 and APP23 mice (Cataldo et al., 2003). Enlarged EEs found in APP23 (Choi et al., 2013) Aβ42 accumulates in endosomal compartments in Tg2576 mice before plaque deposition, and increases with age (Takahashi et al., 2002)
Neuroinflammation and glial phenotypes	Increased astrocytic protein expression and metabolic activity in old Ts65Dn mice (Holtzman et al., 1996; Contestabile et al., 2006) Increased microglial activation in basal forebrain and hippocampus of old Ts65Dn mice (Hunter et al., 2004; Lockrow et al., 2011a)		Astrocytic changes in morphology and increased calcium signaling in *APP* transgenic mice (Takano et al., 2007; Beauquis et al., 2013; Rodríguez-Arellano et al., 2015) Impairments in microglia phagocytosis and increased microglia proliferation around plaques in APP23 and Tg2576 (Frautschy et al., 1998; Krabbe et al., 2013)

*Abbreviations: AChE, acetylcholinesterase; AKT, protein kinase B; BFCN, basal forebrain cholinergic neuron; CA1, Cornu Ammonis area 1; CDK5, cyclin-dependent kinase 5; ChAT, choline acetyltransferase; CTF, C-terminal fragment; EE, early endosome; GSK-3β, glycogen synthase kinase 3β; LC, locus coeruleus; LTP, long-term potentiation; mGluR5, metabotropic glutamate receptor 5; ROS, reactive oxygen species; SDS, sodium-dodecyl sulfate*.

## Modeling amyloid deposition in mice

In contrast to the segmental duplication of tens of endogenous wildtype genes in DS mouse strains, AD models are primarily transgenic lines that overexpress one or more of the human mutant genes that cause FAD. These transgenes usually insert at random sites in the genome and may be driven by artificial promoters (see examples in Table 4), which vary in terms of their spatial and temporal expression patterns, and result in expression at often 5–10 fold compared to endogenous mouse orthologue (Balducci and Forloni, 2011; Hall and Roberson, 2012). Overexpressing wildtype human *APP* or mouse *App* does not result in amyloid deposition (Elder et al., 2010); hence the need to use known AD-causative mutant sequences in transgenic mice.

**Table 4:** **Human *APP* overexpressing transgenic mice referred to in this review (information obtained from Alzforum.org)**.

**Mouse**	**Mutation**	**Promoter**	**Genetic Background**	**References**
APP22	APP751 KM670/671NL (Swedish), V717I (London)	Human THY1	C57BL/6	Sturchler-Pierrat et al., 1997
APP23	APP751 KM670/671NL (Swedish)	Mouse Thy1	C57BL/6	Sturchler-Pierrat et al., 1997
APP(V717I)	APP695 V717I (London)	Mouse Thy1	Originally generated on FVB/N background; available at reMYND as C57BL/6xFVB/N	Moechars et al., 1999
Tg2576	APP695 KM670/671NL (Swedish)	Hamster prion protein	C57BL/6;SJL mixed background	Hsiao et al., 1996
TgCRND8	APP KM670/671NL (Swedish), V717F (Indiana)	Hamster prion protein	C3H/He-C57BL/6 mixed background	Chishti et al., 2001
PDAPP	APP V717F (Indiana)	Human PDGF	C57BL/6 x DBA2	Games et al., 1995

In general, while mutant *APP* transgenic mice develop robust amyloid deposition, synaptotoxic features and memory impairments, none of them reproduces tau-containing neurofibrillary tangles, the hallmark pathology of AD which most closely correlates with dementia (Hall and Roberson, 2012). The combined overexpression of mutant *APP* and mutant human tau is required to reproduce both amyloid and tau pathology, although these tau mutations in humans do not alone cause AD but another form of neurodegeneration, frontotemporal dementia. Mutant *APP* transgenics may be best considered models of APP/Aβ pathology (amyloid deposition) rather than full AD.

## Studying AD-DS phenotypes in mice

In Table 3, we summarize examples of findings that may be informative for AD-DS from different DS (mainly Ts65Dn) mice and examples of AD models (Table 4). With respect to AD, a wide range of mutant *APP* transgenic strains are available in the literature, so we have chosen a few well-known examples [APP22, APP23, APP (V717I), PDAPP, Tg2576, TgCRND8] to illustrate some potential phenotypes of interest. We note that the expression of wildtype mouse APP, and wildtype or mutant human APP protein in these different models can influence amyloid pathology (Kokjohn and Roher, 2009). For example, because of amino acid differences between the two species, mouse APP may be processed with little BACE1 cleavage and so may yield three times less Aβ than wildtype human APP (De Strooper et al., 1995). In addition, the genetic background of AD mouse strains affects a range of APP/Aβ phenotypes, including plaque deposition, APP metabolism, survival, and seizure rates (Carlson et al., 1997; Lehman et al., 2003; Krezowski et al., 2004; Lassalle et al., 2008; Rustay et al., 2010; Jackson et al., 2015). Similarly, phenotypes observed in DS mice may be influenced by genetic background (O'Doherty et al., 2005; Galante et al., 2009; Costa et al., 2010; Deitz and Roper, 2011; Haydar and Reeves, 2012). We consider only *APP* transgenic models of AD, as the other genes used in such models (*PSEN1, PSEN2*, and *MAPT*) are not encoded on Hsa21, and therefore are not directly relevant to AD-DS.

In studying mouse phenotypes to understand AD-DS, we are presented with two key issues. Firstly, we need to test longitudinally DS models to look for changes in older mice that are not apparent early on, and so may indicate aging or neurodegenerative processes rather than neurodevelopmental deficits. Secondly, we need to separate normal aging processes in DS from those connected specifically to AD-DS. The thoughtful use of the increasing range of different mouse models is enabling us to dissect these issues to further our understanding of AD-DS.

A study that has addressed both (1) neurodegenerative vs. neurodevelopmental and (2) normal aging vs. AD phenotypes has been performed in the Ts65Dn mouse. This study concerned the neurodegenerative phenotype loss of basal forebrain cholinergic neurons (BFCNs), and was carried out through an experimental design involving optimal crossing of different mouse models and assessment of the genetically-distinct progeny (Salehi et al., 2006). Firstly, Salehi and colleagues quantified the known loss of BFCNs in Ts65Dn mice, and showed this loss to be progressive, thus an aging or an AD-related phenotype in this DS mouse model. The authors then compared BFCN loss in Ts65Dn and Ts1Cje DS mouse models (Figure 1), and were able to map a dosage-sensitive critical region that had to contain a candidate gene for this phenotype: Ts65Dn mice lose BFCNs but Ts1Cje mice turned out to have no loss compared to wildtype mice. Therefore, the dosage-sensitive gene(s), that when present in three copies is responsible for BFCN loss, must map within the region of trisomy present in Ts65Dn but not in Ts1Cje. A key candidate in this region was the *App* gene. By crossing Ts65Dn mice to heterozygous *App* knockout mice, the authors generated cohorts of progeny that carried the trisomic region with either two or three copies of wildtype *App*. Assessing BFCN loss in these cohorts led to the conclusion that the phenotype arises mainly from having three copies of *App* and, further, that it is associated with impairments in nerve growth factor retrograde transport, linked to early endosomes, which are enlarged (Salehi et al., 2006).

Given the role of *APP* triplication in this phenotype, there is likely a strong link to AD and AD-DS. In people with early AD pathology or mild cognitive impairment, neurofibrillary pathology has been detected in BFCNs (Mesulam et al., 2004; Grudzien et al., 2007), while their loss has been observed in patients with SAD (and other neurodegenerative disorders; Zarow et al., 2003). Interestingly, enlarged early endosomes have been detected in cortical tissues from cognitively intact individuals with mild AD pathology, and in young individuals with DS (under 12 years old), suggesting that endosome enlargement is an early feature in AD pathogenesis (Cataldo et al., 2000).

## DS models in the study of candidate genes influencing AD

As illustrated in Table 1, while people with DS have three copies of *APP* and develop early AD neuropathology, their clinical presentation is variable, suggesting that other genetic and environmental factors influence pathogenesis. In addition to *APP*, many genes on Hsa21 have been studied in the context of neurodegeneration and/or AD, and it is conceivable that a three-copy dose of any of these genes could contribute to disease and dysfunction.

Single gene overexpressing transgenics do not model DS, or AD-DS, but may provide some insights if carefully considered. For example, seizures and neuronal network abnormalities remain challenging areas to investigate but important phenotypes to be explored in DS, AD-DS, and *APP* overexpression models of AD (i.e., which are single gene transgenic models). In SAD, seizures have been associated with early cognitive decline (Vossel et al., 2013), while the incidence of seizures in AD-DS is high and is associated with increased risk of dementia (for example, McCarron et al., 2014). To date, seizure phenotypes and epileptiform activity have been characterized across numerous *APP* transgenic mice (Born, 2015), but it is unclear whether these phenotypes are primarily driven by amyloid overproduction (Mucke and Selkoe, 2012) or are an effect of unphysiological *APP* overexpression during development (Born et al., 2014). Antiepileptic drugs, such as levetiracetam, which improve seizures in DS (Sangani et al., 2010) and in AD (Cumbo and Ligori, 2010), also ameliorate synaptic and memory dysfunctions in APP transgenic mice by suppressing neuronal network dysfunction (Sanchez et al., 2012; Devi and Ohno, 2013).

So, while single gene transgenic models do not model human trisomy 21 or AD because they usually express the gene by many-fold, from ectopic promoters, they offer insights into some of the functional consequences of overexpression, albeit at non-trisomic levels. Table 5 presents a list of Hsa21 gene candidates, in chromosomal order, that have been investigated for overexpression-related phenotypes linked with AD across different mouse, fruitfly, and cellular models. We also compare, where data are available, how related changes in these genes have been explored in humans with AD and/or DS. Making optimal use of mouse genetics, some of the single-gene-overexpressing mouse transgenics have been crossed with AD models, to look for changes in phenotypes that may be informative. For example, crossing an *S100*β overexpression model with the Tg2576 *APP* transgenic mouse generates double mutant progeny with exacerbated cerebral amyloidosis and reactive gliosis. This suggests that increased expression of *S100*β could contribute to AD pathogenesis possibly by promoting amyloidogenic APP processing (Mori et al., 2010).

**Table 5:** **Single gene overexpression models from Hsa21, with relevance to AD phenotypes. Genes are listed in order from centromere to Hsa21q telomere**.

**Hsa21 gene**	**Phenotypes studied in models**	**Phenotypes studied in humans**
*APP*	Please refer to Table 3.	Please refer to Table 1
*SOD1*	*SOD1* overexpression protects against APP-induced lethality in transgenic mice (Carlson et al., 1997)	SOD1 activity positively correlates with levels of memory functioning in DS adults (Zis et al., 2012)
*ITSN1*	Overexpression of *ITSN1* homolog *nla* in combination with *SYNJ1* and *RCAN1* homologs causes impaired vesicle recycling in *Drosophila* (Chang and Min, 2009)	ITSN1 protein (Hunter et al., 2011) and mRNA (Pucharcos et al., 1999) elevated in DS *ITSN1* highly expressed in AD brain (Blalock et al., 2004; Wilmot et al., 2008)
*SYNJ1*	Mice overexpressing *SYNJ1* have deficits in synaptic transmission (Voronov et al., 2008) *SYNJ1* transgenic mice display enlarged endosomes (Cossec et al., 2012)	SYNJ1 levels higher in DS brain tissue compared to controls, and elevated in AD-DS cases (Martin et al., 2014)
*OLIG2*	Neural progenitors from *Olig2*-overexpressing mice exhibit impairments in neural progenitor proliferation (Lu et al., 2012)	SNPs in *OLIG2* associated with psychotic symptoms in AD (Sims et al., 2009)
*RCAN1*	*RCAN1* overexpression in a mouse model causes abnormal tau phosphorylation (Wegiel et al., 2011) In cell models, *RCAN1* overexpression leads to deficits in synaptic transmission (Martin et al., 2012) and promotes neuronal apoptosis (Sun et al., 2011, 2014)	*RCAN1* chronically elevated in AD and DS (Ermak et al., 2001)
*DYRK1A*	*DYRK1A* overexpression linked to tau hyperphosphorylation and increased Aβ production in transgenic mice (Ryoo et al., 2007, 2008) and cellular models (Park et al., 2007; Coutadeur et al., 2015) *Dyrk1a* overexpression causes phosphorylation of PS1, increasing γ-secretase activity in cells and stabilizing γ-secretase complex in mice (Ryu et al., 2010) Mouse *Dyrk1a* overexpression in TgDyrk1A mice results in a significant reduction of *Rest* mRNA (Canzonetta et al., 2008)	DYRK1A increased in the brains of patients with AD (Kimura et al., 2007) and DS (Ryoo et al., 2008) DYRK1A expression in DS brain correlates with 3-repeat tau levels (Shi et al., 2008; Wegiel et al., 2011) Plasma DYRK1A positively correlates with cerebrospinal fluid tau and phospho-tau in AD patients (Janel et al., 2014) Co-localization of DYRK1A with NFTs greater in AD-DS than SAD (Wegiel et al., 2008) REST levels correlate with cognitive preservation and longevity in aging and are downregulated in AD (Lu et al., 2014)
*DSCAM*	Trisomy of *Dscam* in *Drosophila* results in synaptic targeting errors (Cvetkovska et al., 2013)	DSCAM overexpressed in a DS patient, and DSCAM immunoreactivity associated with Aβ plaques in demented DS patients (Saito et al., 2000)
*ETS2*	*Ets2* transgenic mice and fibroblasts overexpressing ETS2 have elevated APP, presenilin1 protein and increased Aβ production (Wolvetang et al., 2003b) *Ets2* overexpression causes apoptosis via caspase 3 activation in primary neuronal cultures (Wolvetang et al., 2003a) and in DS cortical neurons (Helguera et al., 2005)	ETS2 immunoreactivity associated with intracellular Aβ and hyperphosphorylated tau in both AD-DS and sporadic AD brain tissue (Helguera et al., 2005)
*BACE2*	*BACE2* overexpression *in vitro* reduces Aβ levels (Sun et al., 2006) In a mouse model, overexpression of *BACE2* has no effect on Aβ production (Azkona et al., 2010a,b)	*BACE2* polymorphisms may predict age of onset of dementia in DS (Myllykangas et al., 2005; Mok et al., 2014)
		
*ABCG1*	*ABCG1* overexpression stimulates cholesterol efflux *in vitro* (Kim et al., 2007; Tansley et al., 2007) and either reduces (Kim et al., 2007) or increases Aβ production (Tansley et al., 2007), the latter through an increase in APP processing *ABCG1* overexpression in a mouse model has no effect on reference or working memory or synaptic plasticity (Parkinson et al., 2009), nor alters Aβ, APOE nor cholesterol efflux *in vivo* (Burgess et al., 2008)	*ABCG1* gene upregulated in patients with DS (Tansley et al., 2007; Kong et al., 2015) *ABCG1* gene expression unaltered in AD (Tansley et al., 2007)
*CSTB*	*Cstb* overexpression in a mouse model does not induce epileptic activity or a myoclonic seizure phenotype (Brault et al., 2011)	CSTB protein unaltered in DS fetal cerebral cortex (Cheon et al., 2003b).
*SUMO3*	*SUMO3* overexpression in cell culture systems shown to both increase (Dorval et al., 2007) and reduce (Zhang and Sarge, 2008) Aβ levels *SUMO3* overexpression modulates APP processing, increasing the CTF/APP ratio *in vitro* (Dorval et al., 2007)	High molecular weight SUMO3 conjugates decreased in AD brain tissue (Lee et al., 2014)
*S100β*	S100β application results in tau hyperphosphorylation in cultured neural stem cells (Esposito et al., 2008) *S100β* overexpression increases neuronal death and reduces neuronal production in DS stem cells (Lu et al., 2011) *S100β* overexpression in Tg2576 AD mice increases Aβ deposition and BACE1 activity (Mori et al., 2010) Mice overexpressing *S100β* show accelerated signs of aging (Shapiro and Whitaker-Azmitia, 2004) neuropathology (Shapiro et al., 2004) and behavioral deficits (Borella et al., 2003)	*S100β* upregulated in DS and AD (Griffin et al., 1989; Sheng et al., 1994) *S100β* overexpression positively correlates with age in DS patients (Royston et al., 1999)

*SOD1, superoxide dismutase1; ITSN1, intersectin 1; SYNJ1, synaptojanin 1; OLIG2, oligodendrocyte transcription factor 2; RCAN1, regulator of calcineurin 1; DYRK1A, Dual specificity tyrosine-phosphorylation-regulated kinase 1A; DSCAM, Down syndrome cell adhesion molecule; ETS2, V-Ets Avian Erythroblastosis Virus E26 Oncogene Homolog 2; BACE2, beta-site APP cleaving enzyme 2; ABCG1, ATP-binding cassette sub-family G member 1; CSTB, cystatin B; SUMO3, small ubiquitin-like modifier 3; S100β, S100 calcium binding protein β; REST, repressor element-1 silencing transcription factor*.

Other key Hsa21 gene candidates *DYRK1A* and *RCAN1* have been linked to AD pathogenesis through their effects on tau. The toxic neurofibrillary tangles (NFTs) that accumulate in AD are formed of hyperphosphorylated tau protein. Overexpression of *DYRK1A* in transgenic mice resulted in tau hyperphosphorylation (Ryoo et al., 2007, 2008), and DYRK1A has been shown to co-localize with NFTs more frequently in AD-DS brain compared to SAD (Wegiel et al., 2008). Similarly, overexpression of *RCAN1* in a mouse model resulted in abnormal tau hyperphosphorylation (Wegiel et al., 2011). This suggests that the increased expression of *DYRK1A* and *RCAN1* in DS could promote the formation of NFTs, a hallmark feature of AD pathology.

Triplication of Hsa21 genes in DS does not necessarily lead to a 1.5-fold increase (compared to euploid individuals) in their RNA or protein expression. For example, a study in DS fetal cortical tissue revealed multiple Hsa21 proteins in fact expressed at similar or lower levels than in disomic controls (Cheon et al., 2003a,b,c,d). Assessments at transcriptomic and proteomic levels, together with meta-analysis across these studies, provide useful resources for understanding patterns of alteration in gene expression (for example, see Vilardell et al., 2011). As a few of the studies in Table 5 have demonstrated, it is important to verify the effect of trisomy on candidate gene expression, in relevant tissues and contexts, before further characterization of any potential downstream effects of trisomy.

## Prospects for research

Individuals with DS manifest the most common genetic form of AD, and this undoubtedly largely arises from expressing three copies of *APP* (Ness et al., 2012; Hartley et al., 2015). Therefore, studying and modeling this population will assist in understanding the contribution of APP to AD pathogenesis, and evaluating the amyloid cascade hypothesis. However, the variation in clinical presentation of AD-DS shows that many other genetic and environmental factors contribute, almost certainly including protective factors. The thoughtful use of models will thus provide insight into these factors.

To study mouse models of AD-DS, it is critical to dissect neurodevelopmental from neurodegenerative effects (Bothwell and Giniger, 2000; Contestabile et al., 2010). To be of interest for AD-DS, such phenotypes should differ from normal aging in the mouse strain of interest, although this can be difficult to determine, particularly as DS has been characterized as a syndrome of accelerated aging in both clinical (Lott, 2012; Zigman, 2013) and epigenetic terms (Horvath et al., 2015), and because aging remains the clearest non-genetic risk factor for all forms of AD (Fratiglioni, 1996; Bush and Beail, 2004). The longitudinal study of cognitive decline in DS mice poses similar challenges to those in people with DS, and tests need to distinguish between dysfunction due to dementia, as opposed to aging or baseline learning deficits. For example, variations of a learning procedure involving incremental repeated acquisition tasks suggest that declining performances by Ts65Dn mice with age may be due to motor impairments and/or decreased motivation, rather than neurodegenerative-related effects (Sanders et al., 2009). To improve behavioral testing in mouse models of AD-DS, a potential avenue to explore capitalizes on the association of dementia with deficits in episodic memory. The development of tests based on, for example, visuo-spatial data, should therefore highlight age-dependent, dementia-related deficits in mouse models, because they rely on the encoding and binding of information spontaneously, and do not challenge other cognitive domains (Iordanova et al., 2009).

As well as the hypothesis-driven study of AD-DS phenotypes, one of the greatest strengths of working with mouse models is our ability to undertake unbiased hypothesis-generating research, by mapping phenotypes to genomic critical regions using the range of strains now available. These include chromosome-engineered panels of partially trisomic mice (Figure 1) as well as single gene knockout animals, such as the *App*^+∕−^ heterozygous mice, which may be crossed to partially trisomic strains, to generate progeny with altered single gene copy numbers on different trisomic region backgrounds. The cohorts of progeny from these crosses provide ideal groups for testing the contributions of single Hsa21 genes to AD-DS.

Mouse genome engineering continues to offer new models and approaches for teasing apart AD-DS relevant phenotypes, and new strains are being published regularly to help refine experimental strategies. For example, the recent genomically humanized NLF mouse (Saito et al., 2014), which has human amino acid residues at key sites within APP that affect its processing, may yield new insights into the biology of both AD and AD-DS, partly through expressing mutant *APP* at physiological levels. The strategic breeding of new *APP* models with DS segmental trisomies will contribute to determining which phenotypes are downstream of an amyloid cascade. Furthermore, independent study of partial trisomies without three copies of *App* may help tease out effects of other factors, for example oxidative stress, cholesterol metabolism or immune system dysfunction, in the development of dementia (Wiseman et al., 2015).

DS mouse models also give us the flexibility to investigate the effects of potentially dosage-sensitive non-coding regions. For example, microRNAs (miRs)—short (20–23 nucleotide) RNAs that downregulate the transcription of target genes—have increasingly been investigated in AD pathogenesis due to their differential regulation in molecular pathways associated with AD (Veerappan et al., 2013). Hsa21 encodes 29 miRs (MirBase release 21, Griffiths-Jones, 2004), and their potential overexpression in trisomy may contribute to genetic dysregulation relevant to AD-DS. Overexpression of the Hsa21-encoded miR-155 in DS has been reported to increase Aβ production via the downregulation of sorting nexin 27, a membrane-trafficking component found in early endosomes, that modulates γ-secretase activity (Wang et al., 2013, 2014).

Hsa21 also encodes genes involved in post-translational histone modification, including *DYRK1A, ETS2, HMGN1, BRWD1*, and *RUNX1* (Dekker et al., 2014), which may be investigated for their potential roles leading to the aberrant histone modifications observed in AD (Zhang et al., 2012a; Narayan et al., 2015). Histone methylation (specifically H3K4me3) has been shown to correlate highly with genome-wide domains of dysregulated gene expression in DS, which are highly conserved between humans and Ts65Dn mice (Letourneau et al., 2014). DS mouse models therefore model epigenetic structures in humans and may be used to study the effects of its dysregulation in AD-DS.

Finally, mouse model research must be undertaken in parallel with other rapid advances in the AD-DS field. The advent of human induced pluripotent stem (iPS) cells (Hunsberger et al., 2015) for DS provides for the first time a trisomic human *in vitro* model that recapitulates hallmarks of some AD pathology (Shi et al., 2012; Chang et al., 2015; Moore et al., 2015; Murray et al., 2015). The further development of this technology (Hunsberger et al., 2015) will prove valuable to phenotyping and drug target discovery, alongside *in vivo* research and *in vitro* primary cultures from DS mice. An increasing call is being made for partnerships to build up large cohorts of, and biobanks from, people with DS for the systematic longitudinal study of AD-DS progression (Hartley et al., 2015). In-depth phenotypic studies across development with infants and adults with DS are already underway (Wiseman et al., 2015). These will allow greater power to identify biomarkers for the prediction of AD in this large, genetically well-defined population, for example, through plasma (Dekker et al., 2015; Schupf et al., 2015), cerebrospinal fluid (Portelius et al., 2014a,b), and neuroimaging studies (Beacher et al., 2009; Landt et al., 2011; Powell et al., 2014; Sabbagh et al., 2015). Biomarker studies are also being performed in AD models, including at very early phases of Aβ deposition (Maia et al., 2015). Extending these studies to mouse models of DS and AD-DS will contribute to elucidating the genotype-phenotype relationships that ultimately lead to dementia.

### Conflict of interest statement

The authors declare that the research was conducted in the absence of any commercial or financial relationships that could be construed as a potential conflict of interest.

## References

[B1] Adams D. Oliver C. (2010). The relationship between acquired impairments of executive function and behaviour change in adults with Down syndrome. J. Intellect. Disabil. Res. 54, 393–405. 10.1111/j.1365-2788.2010.01271.x 20367747

[B2] Albert M. S. DeKosky S. T. Dickson D. Dubois B. Feldman H. H. Fox N. C.. (2011). The diagnosis of mild cognitive impairment due to Alzheimer's disease: recommendations from the National Institute on Aging-Alzheimer's Association workgroups on diagnostic guidelines for Alzheimer's disease. Alzheimer's Dementia 7, 270–279. 10.1016/j.jalz.2011.03.008 21514249 PMC3312027

[B3] Apelt J. Kumar A. Schliebs R. (2002). Impairment of cholinergic neurotransmission in adult and aged transgenic Tg2576 mouse brain expressing the Swedish mutation of human beta-amyloid precursor protein. Brain Res. 953, 17–30. 10.1016/S0006-8993(02)03262-6 12384234

[B4] Azkona G. Amador-Arjona A. Obradors-Tarragó C. Varea E. Arqué G. Pinacho R.. (2010a). Characterization of a mouse model overexpressing beta-site APP-cleaving enzyme 2 reveals a new role for BACE2. Genes Brain Behav. 9, 160–172. 10.1111/j.1601-183X.2009.00538.x 19840121

[B5] Azkona G. Levannon D. Groner Y. Dierssen M. (2010b). *In vivo* effects of APP are not exacerbated by BACE2 co-overexpression: behavioural characterization of a double transgenic mouse model. Amino Acids 39, 1571–1580. 10.1007/s00726-010-0662-8 20596738

[B6] Bai X. Edden R. A. E. Gao F. Wang G. Wu L. Zhao B.. (2014). Decreased γ-aminobutyric acid levels in the parietal region of patients with Alzheimer's disease. Magn. Reson. Imaging 41, 1326–1331. 10.1002/jmri.24665 24863149 PMC5512098

[B7] Balducci C. Forloni G. (2011). APP transgenic mice: their use and limitations. NeuroMol. Med. 13, 117–137. 10.1007/s12017-010-8141-7 21152995

[B8] Ball S. L. Holland A. J. Treppner P. Watson P. C. Huppert F. A. (2008). Executive dysfunction and its association with personality and behaviour changes in the development of Alzheimer's disease in adults with Down syndrome and mild to moderate learning disabilities. Br. J. Clin. Psychol. 47, 1–29. 10.1348/014466507X230967 17681112

[B9] Basun H. Bogdanovic N. Ingelsson M. Almkvist O. Näslund J. Axelman K.. (2008). Clinical and neuropathological features of the arctic APP gene mutation causing early-onset Alzheimer disease. Arch. Neurol. 65, 499–505. 10.1001/archneur.65.4.499 18413473 PMC2723757

[B10] Beacher F. Daly E. Simmons A. Prasher V. Morris R. Robinson C.. (2009). Alzheimer's disease and Down's syndrome: an *in vivo* MRI study. Psychol. Med. 39, 675–684. 10.1017/S0033291708004054 18667098

[B11] Beauquis J. Pavía P. Pomilio C. Vinuesa A. Podlutskaya N. Galvan V.. (2013). Environmental enrichment prevents astroglial pathological changes in the hippocampus of APP transgenic mice, model of Alzheimer's disease. Exp. Neurol. 239, 28–37. 10.1016/j.expneurol.2012.09.009 23022919

[B12] Belichenko P. V. Masliah E. Kleschevnikov A. M. Villar A. J. Epstein C. J. Salehi A.. (2004). Synaptic structural abnormalities in the Ts65Dn mouse model of Down Syndrome. J. Comp. Neurol. 480, 281–298. 10.1002/cne.20337 15515178

[B13] Bittles A. H. Glasson E. J. (2004). Clinical, social, and ethical implications of changing life expectancy in Down syndrome. Dev. Med. Child Neurol. 46, 282–286. 10.1111/j.1469-8749.2004.tb00483.x 15077706

[B14] Blalock E. M. Geddes J. W. Chen K. C. Porter N. M. Markesbery W. R. Landfield P. W. (2004). Incipient Alzheimer's disease: microarray correlation analyses reveal major transcriptional and tumor suppressor responses. Proc. Natl. Acad. Sci. U.S.A. 101, 2173–2178. 10.1073/pnas.0308512100 14769913 PMC357071

[B15] Boncristiano S. Calhoun M. E. Kelly P. H. Pfeifer M. Bondolfi L. Stalder M.. (2002). Cholinergic changes in the APP23 transgenic mouse model of cerebral amyloidosis. J. Neurosci. 22, 3234–3243. 11943824 10.1523/JNEUROSCI.22-08-03234.2002 PMC6757538

[B16] Borella A. Sumangali R. Ko J. Whitaker-Azmitia P. M. (2003). Characterization of social behaviors and oxytocinergic neurons in the S-100 beta overexpressing mouse model of Down Syndrome. Behav. Brain Res. 141, 229–236. 10.1016/S0166-4328(02)00373-X 12742260

[B17] Born H. A. Kim J. Y. Savjani R. R. Das P. Dabaghian Y. A. Guo Q.. (2014). Genetic suppression of transgenic APP rescues Hypersynchronous network activity in a mouse model of Alzeimer's disease. J. Neurosci. 34, 3826–3840. 10.1523/JNEUROSCI.5171-13.2014 24623762 PMC3951689

[B18] Born H. A. (2015). Seizures in Alzheimer's disease. Neuroscience 286C, 251–263. 10.1016/j.neuroscience.2014.11.051 25484360

[B19] Bothwell M. Giniger E. (2000). Alzheimer's Disease. Cell 102, 271–273. 10.1016/S0092-8674(00)00032-5 10975517

[B20] Braak H. Braak E. (1991). Neuropathological stageing of Alzheimer-related changes. Acta Neuropathol. 82, 239–259. 10.1007/BF00308809 1759558

[B21] Braidy N. Muñoz P. Palacios A. G. Castellano-Gonzalez G. Inestrosa N. C. Chung R. S.. (2012). Recent rodent models for Alzheimer's disease: clinical implications and basic research. J. Neural Transm. 119, 173–195. 10.1007/s00702-011-0731-5 22086139

[B22] Brault V. Duchon A. Romestaing C. Sahun I. Pothion S. Karout M.. (2015). Opposite phenotypes of muscle strength and locomotor function in mouse models of partial trisomy and monosomy 21 for the proximal Hspa13-App region. PLoS Genet. 11:e1005062. 10.1371/journal.pgen.1005062 25803843 PMC4372517

[B23] Brault V. Martin B. Costet N. Bizot J. C. Hérault Y. (2011). Characterization of PTZ-induced seizure susceptibility in a down syndrome mouse model that overexpresses CSTB. PLoS ONE 6:e27845. 10.1371/journal.pone.0027845 22140471 PMC3227573

[B24] Brault V. Pereira P. Duchon A. Hérault Y. (2006). Modeling chromosomes in mouse to explore the function of genes, genomic disorders, and chromosomal organization. PLoS Genet. 2:e86. 10.1371/journal.pgen.0020086 16839184 PMC1500809

[B25] Burgess B. L. Parkinson P. F. Racke M. M. Hirsch-Reinshagen V. Fan J. Wong C.. (2008). ABCG1 influences the brain cholesterol biosynthetic pathway but does not affect amyloid precursor protein or apolipoprotein E metabolism *in vivo*. J. Lipid Res. 49, 1254–1267. 10.1194/jlr.M700481-JLR200 18314463

[B26] Burns A. Jacoby R. Levy R. (1990). Psychiatric phenomena in Alzheimer's disease. I Disorders of thought content. Br. J. Psychiatry 157, 72–76. 10.1192/bjp.157.1.72 2397365

[B27] Bush A. Beail N. (2004). Risk factors for dementia in people with down syndrome: issues in assessment and diagnosis. Am. J. Ment. Retard. 109, 83–97. 10.1352/0895-8017(2004)109<83:RFFDIP>2.0.CO;2 15000668

[B28] Cabrejo L. Guyant-Maréchal L. Laquerrière A. Vercelletto M. De la Fournière F. Thomas-Antérion C.. (2006). Phenotype associated with APP duplication in five families. Brain 129, 2966–2976. 10.1093/brain/awl237 16959815

[B29] Canzonetta C. Mulligan C. Deutsch S. Ruf S. O'Doherty A. Lyle R.. (2008). DYRK1A-dosage imbalance perturbs NRSF/REST levels, deregulating pluripotency and embryonic stem cell fate in Down syndrome. Am. J. Hum. Genet. 83, 388–400. 10.1016/j.ajhg.2008.08.012 18771760 PMC2556438

[B30] Carlson G. A. Borchelt D. R. Dake A. Turner S. Danielson V. Coffin J. D.. (1997). Genetic modification of the phenotypes produced by amyloid precursor protein overexpression in transgenic mice. Hum. Mol. Genet. 6, 1951–1959. 10.1093/hmg/6.11.1951 9302276

[B31] Casanova M. F. Walker L. C. Whitehouse P. J. Price D. L. (1985). Abnormalities of the nucleus basalis in Down's syndrome. Ann. Neurol. 18, 310–313. 10.1002/ana.410180306 2932050

[B32] Castellani R. J. Perry G. (2014). The complexities of the pathology-pathogenesis relationship in Alzheimer disease. Biochem. Pharmacol. 88, 671–676. 10.1016/j.bcp.2014.01.009 24447936

[B33] Cataldo A. Rebeck G. W. Ghetri B. Hulette C. Lippa C. Van Broeckhoven C.. (2001). Endocytic disturbances distinguish among subtypes of Alzheimer's disease and related disorders. Ann. Neurol. 50, 661–665. 10.1002/ana.1254 11706973

[B34] Cataldo A. M. Barnett J. L. Pieroni C. Nixon R. A. (1997). Increased Neuronal Endocytosis and Protease Delivery to Early Endosomes in Sporadic Alzheimer's Disease: neuropathologic Evidence for a Mechanism of Increased beta -Amyloidogenesis. J. Neurosci. 17, 6142–6151. 9236226 10.1523/JNEUROSCI.17-16-06142.1997 PMC6568334

[B35] Cataldo A. M. Petanceska S. Peterhoff C. M. Terio N. B. Epstein C. J. Villar A.. (2003). App Gene dosage modulates endosomal abnormalities of Alzheimer's Disease in a segmental Trisomy 16 mouse model of down syndrome. J. Neurosci. 23, 6788–6792. 12890772 10.1523/JNEUROSCI.23-17-06788.2003 PMC6740714

[B36] Cataldo A. M. Peterhoff C. M. Troncoso J. C. Gomez-Isla T. Hyman B. T. Nixon R. A. (2000). Endocytic pathway abnormalities precede amyloid beta deposition in sporadic Alzheimer's disease and Down syndrome: differential effects of APOE genotype and presenilin mutations. Am. J. Pathol. 157, 277–286. 10.1016/S0002-9440(10)64538-5 10880397 PMC1850219

[B37] Chang C.-Y. Chen S.-M. Lu H.-E. Lai S.-M. Lai P.-S. Shen P.-W.. (2015). N-butylidenephthalide attenuates Alzheimer's Disease-like Cytopathy in down syndrome induced pluripotent stem cell-derived neurons. Sci. Rep. 5, 8744. 10.1038/srep08744 25735452 PMC4348654

[B38] Chang K. T. Min K. T. (2009). Upregulation of three Drosophila homologs of human chromosome 21 genes alters synaptic function: implications for Down syndrome. Proc. Natl. Acad. Sci. U.S.A. 106, 17117–17122. 10.1073/pnas.0904397106 19805187 PMC2761307

[B39] Cheon M. S. Bajo M. Kim S. H. Claudio J. O. Stewart A. K. Patterson D.. (2003a). Protein levels of genes encoded on chromosome 21 in fetal Down syndrome brain: challenging the gene dosage effect hypothesis (Part II). Amino Acids 24, 119–125. 10.1007/s00726-002-0337-1 12624743

[B40] Cheon M. S. Kim S. H. Ovod V. Kopitar Jerala N. Morgan J. I. Hatefi Y.. (2003b). Protein levels of genes encoded on chromosome 21 in fetal Down syndrome brain: challenging the gene dosage effect hypothesis (Part III). Amino Acids 24, 127–134. 10.1007/s00726-002-0340-6 12624744

[B41] Cheon M. S. Kim S. H. Yaspo M.-L. Blasi F. Aoki Y. Melen K.. (2003c). Protein levels of genes encoded on chromosome 21 in fetal Down syndrome brain: challenging the gene dosage effect hypothesis (Part I). Amino Acids 24, 111–117. 10.1007/s00726-002-0336-2 12624742

[B42] Cheon M. S. Shim K. S. Kim S. H. Hara A. Lubec G. (2003d). Protein levels of genes encoded on chromosome 21 in fetal Down syndrome brain: challenging the gene dosage effect hypothesis (Part IV). Amino Acids 25, 41–47. 10.1007/s00726-003-0009-9 12836057

[B43] Chishti M. A. Yang D. S. Janus C. Phinney A. L. Horne P. Pearson J.. (2001). Early-onset amyloid deposition and cognitive deficits in transgenic mice expressing a double mutant form of amyloid precursor protein 695. J. Biol. Chem. 276, 21562–21570. 10.1074/jbc.M100710200 11279122

[B44] Choi J. H. K. Berger J. D. Mazzella M. J. Morales-Corraliza J. Cataldo A. M. Nixon R. A.. (2009). Age-dependent dysregulation of brain amyloid precursor protein in the Ts65Dn Down syndrome mouse model. J. Neurochem. 110, 1818–1827. 10.1111/j.1471-4159.2009.06277.x 19619138 PMC2744432

[B45] Choi J. H. K. Kaur G. Mazzella M. J. Morales-Corraliza J. Levy E. Mathews P. M. (2013). Early endosomal abnormalities and cholinergic neuron degeneration in Amyloid-β protein precursor transgenic mice. J. Alzheimers Dis. 34, 691–700. 10.3233/JAD-122143 23254640 PMC3616896

[B46] Contestabile A. Benfenati F. Gasparini L. (2010). Communication breaks-Down: from neurodevelopment defects to cognitive disabilities in Down syndrome. Prog. Neurobiol. 91, 1–22. 10.1016/j.pneurobio.2010.01.003 20097253

[B47] Contestabile A. Fila T. Bartesaghi R. Contestabile A. Ciani E. (2006). Choline acetyltransferase activity at different ages in brain of Ts65Dn mice, an animal model for Down's syndrome and related neurodegenerative diseases. J. Neurochem. 97, 515–526. 10.1111/j.1471-4159.2006.03769.x 16539660

[B48] Cooper J. D. Salehi A. Delcroix J. D. Howe C. L. Belichenko P. V. Chua-Couzens J.. (2001). Failed retrograde transport of NGF in a mouse model of Down's syndrome: reversal of cholinergic neurodegenerative phenotypes following NGF infusion. Proc. Natl. Acad. Sci. U.S.A. 98, 10439–10444. 10.1073/pnas.181219298 11504920 PMC56979

[B49] Coppus A. Evenhuis H. Verberne G.-J. Visser F. van Gool P. Eikelenboom P.. (2006). Dementia and mortality in persons with Down's syndrome. J. Intellect. Disabil. Res. 50, 768–777. 10.1111/j.1365-2788.2006.00842.x 16961706

[B50] Coppus A. M. W. Evenhuis H. M. Verberne G.-J. Visser F. E. Oostra B. A. Eikelenboom P.. (2008). Survival in elderly persons with Down syndrome. J. Am. Geriatr. Soc. 56, 2311–2316. 10.1111/j.1532-5415.2008.01999.x 19093931

[B51] Cossec J. C. Lavaur J. Berman D. E. Rivals I. Hoischen A. Stora S.. (2012). Trisomy for synaptojanin1 in down syndrome is functionally linked to the enlargement of early endosomes. Hum. Mol. Genet. 21, 3156–3172. 10.1093/hmg/dds142 22511594 PMC3384382

[B52] Costa A. C. S. Stasko M. R. Schmidt C. Davisson M. T. (2010). Behavioral validation of the Ts65Dn mouse model for Down syndrome of a genetic background free of the retinal degeneration mutation Pde6brd1. Behav. Brain Res. 206, 52–62. 10.1016/j.bbr.2009.08.034 19720087 PMC2783207

[B53] Coutadeur S. Benyamine H. Delalonde L. de Oliveira C. Leblond B. Foucourt A.. (2015). A novel DYRK1A (Dual specificity tyrosine phosphorylation-regulated kinase 1A) inhibitor for the treatment of Alzheimer's disease: effect on Tau and amyloid pathologies *in vitro*. J. Neurochem. 133, 440–451. 10.1111/jnc.13018 25556849

[B54] Crews L. Rockenstein E. Masliah E. (2010). APP transgenic modeling of Alzheimer's disease: mechanisms of neurodegeneration and aberrant neurogenesis. Brain Struct. Funct. 214, 111–126. 10.1007/s00429-009-0232-6 20091183 PMC2847155

[B55] Cumbo E. Ligori L. D. (2010). Levetiracetam, lamotrigine, and phenobarbital in patients with epileptic seizures and Alzheimer's disease. Epilepsy Behav. 17, 461–466. 10.1016/j.yebeh.2010.01.015 20188634

[B56] Cvetkovska V. Hibbert A. D. Emran F. Chen B. E. (2013). Overexpression of Down syndrome cell adhesion molecule impairs precise synaptic targeting. Nat. Neurosci. 16, 677–682. 10.1038/nn.3396 23666178 PMC3954815

[B57] Das I. Reeves R. H. (2011). The use of mouse models to understand and improve cognitive deficits in Down syndrome. Dis. Model. Mech. 4, 596–606. 10.1242/dmm.007716 21816951 PMC3180223

[B58] Davisson M. T. Schmidt C. Reeves R. H. Irving N. G. Akeson E. C. Harris B. S.. (1993). Segmental trisomy as a mouse model for Down syndrome. Prog. Clin. Biol. Res. 384, 117–133. 8115398

[B59] De Simone R. Puig X. S. Gelisse P. Crespel A. Genton P. Gélisse P. (2010). Senile myoclonic epilepsy: delineation of a common condition associated with Alzheimer's disease in Down syndrome. Seizure 19, 383–389. 10.1016/j.seizure.2010.04.008 20598585

[B60] De Strooper B. Simons M. Multhaup G. Van Leuven F. Beyreuther K. Dotti C. G. (1995). Production of intracellular amyloid-containing fragments in hippocampal neurons expressing human amyloid precursor protein and protection against amyloidogenesis by subtle amino acid substitutions in the rodent sequence. EMBO J. 14, 4932–4938. 7588622 10.1002/j.1460-2075.1995.tb00176.x PMC394596

[B61] Deitz S. L. Roper R. J. (2011). Trisomic and allelic differences influence phenotypic variability during development of Down syndrome mice. Genetics 189, 1487–1495. 10.1534/genetics.111.131391 21926299 PMC3241431

[B62] Dekker A. D. Coppus A. M. W. Vermeiren Y. Aerts T. van Duijn C. M. Kremer B. P.. (2015). Serum MHPG strongly predicts conversion to Alzheimer's disease in behaviorally characterized subjects with Down syndrome. J. Alzheimers Dis. 43, 871–891. 10.3233/JAD-140783 25125467

[B63] Dekker A. D. De Deyn P. P. Rots M. G. (2014). Epigenetics: the neglected key to minimize learning and memory deficits in Down syndrome. Neurosci. Biobehav. Rev. 45, 72–84. 10.1016/j.neubiorev.2014.05.004 24858130

[B64] Devenny D. A. Zimmerli E. J. Kittler P. Krinsky-McHale S. J. (2002). Cued recall in early-stage dementia in adults with Down's syndrome. J. Intellect. Disabil. Res. 46, 472–483. 10.1046/j.1365-2788.2002.00417.x 12354318

[B65] Devi L. Ohno M. (2013). Effects of levetiracetam, an antiepileptic drug, on memory impairments associated with aging and Alzheimer's disease in mice. Neurobiol. Learn. Mem. 102, 7–11. 10.1016/j.nlm.2013.02.001 23416036

[B66] Di Domenico F. Pupo G. Mancuso C. Barone E. Paolini F. Arena A.. (2015). Bach1 overexpression in Down syndrome correlates with the alteration of the HO-1/BVR-a system: insights for transition to Alzheimer's disease. J. Alzheimers Dis. 44, 1107–1120. 10.3233/JAD-141254 25391381 PMC4677575

[B67] Di Domenico F. Pupo G. Tramutola A. Giorgi A. Schininà M. E. Coccia R.. (2014). Redox proteomics analysis of HNE-modified proteins in Down syndrome brain: clues for understanding the development of Alzheimer disease. Free Radic. Biol. Med. 71, 270–280. 10.1016/j.freeradbiomed.2014.03.027 24675226 PMC4686229

[B68] Dierssen M. Benavides-Piccione R. Martínez-Cué C. Estivill X. Flórez J. Elston G. N.. (2003). Alterations of neocortical pyramidal cell phenotype in the Ts65Dn mouse model of Down syndrome: effects of environmental enrichment. Cereb. Cortex 13, 758–764. 10.1093/cercor/13.7.758 12816891

[B69] Dierssen M. Herault Y. Estivill X. (2009). Aneuploidy: from a physiological mechanism of variance to Down syndrome. Physiol. Rev. 89, 887–920. 10.1152/physrev.00032.2007 19584316

[B70] Dorval V. Mazzella M. J. Mathews P. M. Hay R. T. Fraser P. E. (2007). Modulation of Abeta generation by small ubiquitin-like modifiers does not require conjugation to target proteins. Biochem. J. 404, 309–316. 10.1042/bj20061451 17346237 PMC1868795

[B71] Downes E. C. Robson J. Grailly E. Abdel-All Z. Xuereb J. Brayne C.. (2008). Loss of synaptophysin and synaptosomal-associated protein 25-kDa (SNAP-25) in elderly Down syndrome individuals. Neuropathol. Appl. Neurobiol. 34, 12–22. 10.1111/j.1365-2990.2007.00899.x 18005332

[B72] Duchon A. Raveau M. Chevalier C. Nalesso V. Sharp A. J. Herault Y. (2011). Identification of the translocation breakpoints in the Ts65Dn and Ts1Cje mouse lines: relevance for modeling Down syndrome. Mamm. Genome 22, 674–684. 10.1007/s00335-011-9356-0 21953411 PMC3224224

[B73] Eckert A. Schulz K. L. Rhein V. Götz J. (2010). Convergence of amyloid-β and tau pathologies on mitochondria *in vivo*. Mol. Neurobiol. 41, 107–114. 10.1007/s12035-010-8109-5 20217279 PMC2876263

[B74] Elder G. A. Gama Sosa M. A. De Gasperi R. (2010). Transgenic mouse models of Alzheimer's disease. Mt. Sinai J. Med. 77, 69–81. 10.1002/msj.20159 20101721 PMC2925685

[B75] Ermak G. Morgan T. E. Davies K. J. (2001). Chronic overexpression of the calcineurin inhibitory gene DSCR1 (Adapt78) is associated with Alzheimer's disease. J. Biol. Chem. 276, 38787–38794. 10.1074/jbc.M102829200 11483593

[B76] Esposito G. Scuderi C. Lu J. Savani C. De Filippis D. Iuvone T.. (2008). S100B induces tau protein hyperphosphorylation *via* Dickopff-1 up-regulation and disrupts the Wnt pathway in human neural stem cells. J. Cell. Mol. Med. 12, 914–927. 10.1111/j.1582-4934.2008.00159.x 18494933 PMC3538024

[B77] Fortress A. M. Hamlett E. D. Vazey E. M. Aston-Jones G. Cass W. A. Boger H. A.. (2015). Designer receptors enhance memory in a mouse model of down syndrome. J. Neurosci. 35, 1343–1353. 10.1523/JNEUROSCI.2658-14.2015 25632113 PMC4308587

[B78] Francis B. M. Yang J. Hajderi E. Brown M. E. Michalski B. McLaurin J.. (2012). Reduced Tissue Levels of Noradrenaline Are Associated with Behavioral Phenotypes of the TgCRND8 Mouse Model of Alzheimer's Disease. Neuropsychopharmacology 37, 1934–1944. 10.1038/npp.2012.40 22491352 PMC3376325

[B79] Fratiglioni L. (1996). Epidemiology of Alzheimer's disease and current possibilities for prevention. Acta Neurol. Scand. Suppl. 165, 33–40. 10.1111/j.1600-0404.1996.tb05870.x 8740987

[B80] Frautschy S. A. Yang F. Irrizarry M. Hyman B. Saido T. C. Hsiao K.. (1998). Microglial response to amyloid plaques in APPsw transgenic mice. Am. J. Pathol. 152, 307–317. 9422548 PMC1858113

[B81] Galante M. Jani H. Vanes L. Daniel H. Fisher E. M. C. Tybulewicz V. L. J.. (2009). Impairments in motor coordination without major changes in cerebellar plasticity in the Tc1 mouse model of Down syndrome. Hum. Mol. Genet. 18, 1449–1463. 10.1093/hmg/ddp055 19181682 PMC2664148

[B82] Games D. Adams D. Alessandrini R. Barbour R. Berthelette P. Blackwell C.. (1995). Alzheimer-type neuropathology in transgenic mice overexpressing V717F beta-amyloid precursor protein. Nature 373, 523–527. 10.1038/373523a0 7845465

[B83] Ghezzo A. Salvioli S. Solimando M. C. Palmieri A. Chiostergi C. Scurti M.. (2014). Age-related changes of adaptive and neuropsychological features in persons with Down Syndrome. PLoS ONE 9:e113111. 10.1371/journal.pone.0113111 25419980 PMC4242614

[B84] Glenner G. G. Wong C. W. (1984). Alzheimer's disease: initial report of the purification and characterization of a novel cerebrovascular amyloid protein. Biochem. Biophys. Res. Commun. 120, 885–890. 6375662 10.1016/s0006-291x(84)80190-4

[B85] Goedert M. Spillantini M. G. Cairns N. J. Crowther R. A. (1992). Tau proteins of Alzheimer paired helical filaments: abnormal phosphorylation of all six brain isoforms. Neuron 8, 159–168. 10.1016/0896-6273(92)90117-V 1530909

[B86] Gómez-Isla T. Hollister R. West H. Mui S. Growdon J. H. Petersen R. C.. (1997). Neuronal loss correlates with but exceeds neurofibrillary tangles in Alzheimer's disease. Ann. Neurol. 41, 17–24. 10.1002/ana.410410106 9005861

[B87] Grabowski T. J. Cho H. S. Vonsattel J. P. Rebeck G. W. Greenberg S. M. (2001). Novel amyloid precursor protein mutation in an Iowa family with dementia and severe cerebral amyloid angiopathy. Ann. Neurol. 49, 697–705. 10.1002/ana.1009 11409420

[B88] Gribble S. M. Wiseman F. K. Clayton S. Prigmore E. Langley E. Yang F.. (2013). Massively parallel sequencing reveals the complex structure of an irradiated human chromosome on a mouse background in the Tc1 model of Down syndrome. PLoS ONE 8:e60482. 10.1371/journal.pone.0060482 23596509 PMC3626651

[B89] Griffin W. S. Stanley L. C. Ling C. White L. MacLeod V. Perrot L. J.. (1989). Brain interleukin 1 and S-100 immunoreactivity are elevated in Down syndrome and Alzheimer disease. Proc. Natl. Acad. Sci. U.S.A. 86, 7611–7615. 10.1073/pnas.86.19.7611 2529544 PMC298116

[B90] Griffiths-Jones S. (2004). The microRNA Registry. Nucleic Acids Res. 32, D109–D111. 10.1093/nar/gkh023 14681370 PMC308757

[B91] Grudzien A. Shaw P. Weintraub S. Bigio E. Mash D. C. Mesulam M. M. (2007). Locus coeruleus neurofibrillary degeneration in aging, mild cognitive impairment and early Alzheimer's disease. Neurobiol. Aging 28, 327–335. 10.1016/j.neurobiolaging.2006.02.007 16574280

[B92] Guyant-Marechal I. Berger E. Laquerrière A. Rovelet-Lecrux A. Viennet G. Frebourg T.. (2008). Intrafamilial diversity of phenotype associated with app duplication. Neurology 71, 1925–1926. 10.1212/01.wnl.0000339400.64213.56 19047566

[B93] Haas M. A. Bell D. Slender A. Lana-Elola E. Watson-Scales S. Fisher E. M. C. (2013). Alterations to dendritic spine morphology, but not dendrite patterning, of cortical projection neurons in Tc1 and Ts1Rhr mouse models of Down syndrome. PLoS ONE 8:e78561 10.1371/journal.pone.0078561 24205261 PMC3813676

[B94] Hall A. M. Roberson E. D. (2012). Mouse models of Alzheimer's disease. Brain Res. Bull. 88, 3–12. 10.1016/j.brainresbull.2011.11.017 22142973 PMC3546481

[B95] Hardy J. Selkoe D. J. (2002). The amyloid hypothesis of Alzheimer's disease: progress and problems on the road to therapeutics. Science 297, 353–356. 10.1126/science.1072994 12130773

[B96] Hardy J. A. Higgins G. A. (1992). Alzheimer's disease: the amyloid cascade hypothesis. Science 256, 184–185. 10.1126/science.1566067 1566067

[B97] Hartley D. Blumenthal T. Carrillo M. DiPaolo G. Esralew L. Gardiner K.. (2015). Down syndrome and Alzheimer's disease: common pathways, common goals. Alzheimers Dementia 11, 700–709. 10.1016/j.jalz.2014.10.007 25510383 PMC4817997

[B98] Hashimoto Y. Matsuoka M. (2014). A mutation protective against Alzheimer's disease renders amyloid β precursor protein incapable of mediating neurotoxicity. J. Neurochem. 130, 291–300. 10.1111/jnc.12717 24646423

[B99] Hattori M. Fujiyama A. Taylor T. D. Watanabe H. Yada T. Park H. S.. (2000). The DNA sequence of human chromosome 21. Nature 405, 311–319. 10.1038/35012518 10830953

[B100] Haydar T. F. Reeves R. H. (2012). Trisomy 21 and early brain development. Trends Neurosci. 35, 81–91. 10.1016/j.tins.2011.11.001 22169531 PMC3273608

[B101] Helguera P. Pelsman A. Pigino G. Wolvetang E. Head E. Busciglio J. (2005). ets-2 promotes the activation of a mitochondrial death pathway in Down's syndrome neurons. J. Neurosci. 25, 2295–2303. 10.1523/jneurosci.5107-04.2005 15745955 PMC6726094

[B102] Herault Y. Lopes P. P. Magnol L. Sahun I. Duchon A. Prandini P. (2009). Tackling the complexity of the genotype–phenotype relationship in the Down syndrome with the mouse aneuploidy zoo: a resource of new models to study aneuploidies involving human chromosome 21, in The American Society of Human Genetics 59th Annual Meeting. (Honolulu HI).

[B103] Hernández-González S. Ballestín R. López-Hidalgo R. Gilabert-Juan J. Blasco-Ibáñez J. M. Crespo C.. (2015). Altered distribution of hippocampal interneurons in the murine Down Syndrome Model Ts65Dn. Neurochem. Res. 40, 151–164. 10.1007/s11064-014-1479-8 25399236

[B104] Holland A. J. Hon J. Huppert F. A. Stevens F. Watson P. (1998). Population-based study of the prevalence and presentation of dementia in adults with Down's syndrome. Br. J. Psychiatry 172, 493–498. 9828989 10.1192/bjp.172.6.493

[B105] Holtzman D. M. Santucci D. Kilbridge J. Chua-Couzens J. Fontana D. J. Daniels S. E.. (1996). Developmental abnormalities and age-related neurodegeneration in a mouse model of Down syndrome. Proc. Natl. Acad. Sci. U.S.A. 93, 13333–13338. 10.1073/pnas.93.23.13333 8917591 PMC24093

[B106] Hooli B. V. Mohapatra G. Mattheisen M. Parrado A. R. Roehr J. T. Shen Y.. (2012). Role of common and rare APP DNA sequence variants in Alzheimer disease. Neurology 78, 1250–1257. 10.1212/WNL.0b013e3182515972 22491860 PMC3324321

[B107] Hoozemans J. J. M. Rozemuller A. J. M. van Haastert E. S. Eikelenboom P. van Gool W. A. (2011). Neuroinflammation in Alzheimer's disease wanes with age. J. Neuroinflammation 8:171. 10.1186/1742-2094-8-171 22152162 PMC3248382

[B108] Horvath S. Garagnani P. Bacalini M. G. Pirazzini C. Salvioli S. Gentilini D.. (2015). Accelerated epigenetic aging in Down syndrome. Aging Cell 14, 491–495. 10.1111/acel.12325 25678027 PMC4406678

[B109] Hsiao K. Chapman P. Nilsen S. Eckman C. Harigaya Y. Younkin S.. (1996). Correlative memory deficits, Abeta elevation, and amyloid plaques in transgenic mice. Science 274, 99–102. 8810256 10.1126/science.274.5284.99

[B110] Hunsberger J. Efthymiou A. G. Malik N. Behl M. Mead I. L. Zeng X.. (2015). Induced pluripotent stem cell models to enable *in vitro* models for screening in the CNS. Stem Cells Dev. 24, 1852–1864. 10.1089/scd.2014.0531 25794298 PMC4533087

[B111] Hunter C. L. Bimonte-Nelson H. A. Nelson M. Eckman C. B. Granholm A.-C. (2004). Behavioral and neurobiological markers of Alzheimer's disease in Ts65Dn mice: effects of estrogen. Neurobiol. Aging 25, 873–884. 10.1016/j.neurobiolaging.2003.10.010 15212841

[B112] Hunter C. L. Isacson O. Nelson M. Bimonte-Nelson H. Seo H. Lin L.. (2003). Regional alterations in amyloid precursor protein and nerve growth factor across age in a mouse model of Down's syndrome. Neurosci. Res. 45, 437–445. 10.1016/S0168-0102(03)00005-1 12657457

[B113] Hunter M. P. Nelson M. Kurzer M. Wang X. Kryscio R. J. Head E.. (2011). Intersectin 1 contributes to phenotypes *in vivo*: implications for Down's syndrome. Neuroreport 22, 767–772. 10.1097/WNR.0b013e32834ae348 21876463 PMC3339866

[B114] Ingelsson M. Fukumoto H. Newell K. L. Growdon J. H. Hedley-Whyte E. T. Frosch M. P.. (2004). Early Abeta accumulation and progressive synaptic loss, gliosis, and tangle formation in AD brain. Neurology 62, 925–931. 10.1212/01.WNL.0000115115.98960.37 15037694

[B115] Iordanova M. D. Burnett D. J. Aggleton J. P. Good M. Honey R. C. (2009). The role of the hippocampus in mnemonic integration and retrieval: complementary evidence from lesion and inactivation studies. Eur. J. Neurosci. 30, 2177–2189. 10.1111/j.1460-9568.2009.07010.x 20128853

[B116] Itagaki S. McGeer P. L. Akiyama H. Zhu S. Selkoe D. (1989). Relationship of microglia and astrocytes to amyloid deposits of Alzheimer disease. J. Neuroimmunol. 24, 173–182. 10.1016/0165-5728(89)90115-X 2808689

[B117] Iwatsubo T. Mann D. M. Odaka A. Suzuki N. Ihara Y. (1995). Amyloid beta protein (A beta) deposition: a beta 42(43) precedes A beta 40 in Down syndrome. Ann. Neurol. 37, 294–299. 10.1002/ana.410370305 7695229

[B118] Jackson H. M. Onos K. D. Pepper K. W. Graham L. C. Akeson E. C. Byers C.. (2015). DBA/2J genetic background exacerbates spontaneous lethal seizures but lessens amyloid deposition in a mouse model of Alzheimer's disease. PLoS ONE 10:e0125897. 10.1371/journal.pone.0125897 25933409 PMC4416920

[B119] Janel N. Sarazin M. Corlier F. Corne H. de Souza L. C. Hamelin L.. (2014). Plasma DYRK1A as a novel risk factor for Alzheimer's disease. Transl. Psychiatry 4:e425. 10.1038/tp.2014.61 25116835 PMC4150238

[B120] Jellinger K. A. Lauda F. Attems J. (2007). Sporadic cerebral amyloid angiopathy is not a frequent cause of spontaneous brain hemorrhage. Eur. J. Neurol. 14, 923–928. 10.1111/j.1468-1331.2007.01880.x 17662016

[B121] Jensen K. M. Bulova P. D. (2014). Managing the care of adults with Down's syndrome. BMJ 349:g5596. 10.1136/bmj.g5596 25269800

[B122] Jiang Y. Mullaney K. A. Peterhoff C. M. Che S. Schmidt S. D. Boyer-Boiteau A.. (2010). Alzheimer's-related endosome dysfunction in Down syndrome is Abeta-independent but requires APP and is reversed by BACE-1 inhibition. Proc. Natl. Acad. Sci. U.S.A. 107, 1630–1635. 10.1073/pnas.0908953107 20080541 PMC2824382

[B123] Kasuga K. Shimohata T. Nishimura A. Shiga A. Mizuguchi T. Tokunaga J.. (2009). Identification of independent APP locus duplication in Japanese patients with early-onset Alzheimer disease. J. Neurol. Neurosurg. Psychiatry 80, 1050–1052. 10.1136/jnnp.2008.161703 19684239

[B124] Kerbler G. M. Fripp J. Rowe C. C. Villemagne V. L. Salvado O. Rose S.. (2015). Basal forebrain atrophy correlates with amyloid β burden in Alzheimer's disease. NeuroImage Clin. 7, 105–113. 10.1016/j.nicl.2014.11.015 25610772 PMC4299972

[B125] Kern D. S. Maclean K. N. Jiang H. Synder E. Y. Sladek J. R. Bjugstad K. B. (2011). Neural stem cells reduce hippocampal tau and reelin accumulation in aged Ts65Dn Down syndrome mice. Cell Transplant. 20, 371–379. 10.3727/096368910X528085 20875225

[B126] Kim W. S. Rahmanto A. S. Kamili A. Rye K. A. Guillemin G. J. Gelissen I. C.. (2007). Role of ABCG1 and ABCA1 in regulation of neuronal cholesterol efflux to apolipoprotein E discs and suppression of amyloid-beta peptide generation. J. Biol. Chem. 282, 2851–2861. 10.1074/jbc.M607831200 17121837

[B127] Kimura R. Kamino K. Yamamoto M. Nuripa A. Kida T. Kazui H.. (2007). The DYRK1A gene, encoded in chromosome 21 Down syndrome critical region, bridges between beta-amyloid production and tau phosphorylation in Alzheimer disease. Hum Mol Genet. 16, 15–23. 10.1093/hmg/ddl437 17135279

[B128] Kokjohn T. A. Roher A. E. (2009). Amyloid precursor protein transgenic mouse models and Alzheimer's disease: understanding the paradigms, limitations, and contributions. Alzheimers Dementia 5, 340–347. 10.1016/j.jalz.2009.03.002 19560104 PMC2704491

[B129] Kong X. D. Liu N. Xu X. J. Zhao Z. H. Jiang M. (2015). Screening of human chromosome 21 genes in the dorsolateral prefrontal cortex of individuals with Down syndrome. Mol. Med. Rep. 11, 1235–1239. 10.3892/mmr.2014.2855 25370074

[B130] Korbel J. O. Tirosh-Wagner T. Urban A. E. Chen X.-N. Kasowski M. Dai L.. (2009). The genetic architecture of Down syndrome phenotypes revealed by high-resolution analysis of human segmental trisomies. Proc. Natl. Acad. Sci. U.S.A. 106, 12031–12036. 10.1073/pnas.0813248106 19597142 PMC2709665

[B131] Krabbe G. Halle A. Matyash V. Rinnenthal J. L. Eom G. D. Bernhardt U.. (2013). Functional impairment of microglia coincides with Beta-amyloid deposition in mice with Alzheimer-like pathology. PLoS ONE 8:e60921. 10.1371/journal.pone.0060921 23577177 PMC3620049

[B132] Krezowski J. Knudson D. Ebeling C. Pitstick R. Giri R. K. Schenk D.. (2004). Identification of loci determining susceptibility to the lethal effects of amyloid precursor protein transgene overexpression. Hum. Mol. Genet. 13, 1989–1997. 10.1093/hmg/ddh210 15254013

[B133] Krinsky-McHale S. J. Devenny D. A. Gu H. Jenkins E. C. Kittler P. Murty V. V.. (2008). Successful aging in a 70-year-old man with down syndrome: a case study. Intellect. Dev. Disabil. 46, 215–228. 10.1352/2008.46:215-228 18578579

[B134] Krinsky-McHale S. J. Devenny D. A. Silverman W. P. (2002). Changes in explicit memory associated with early dementia in adults with Down's syndrome. J. Intellect. Disabil. Res. 46, 198–208. 10.1046/j.1365-2788.2002.00365.x 11896805

[B135] Krinsky-McHale S. J. Devenny D. A. Sersen G. Silverman W. P. (2000). Sequence of cognitive decline in dementia in adults with Down's syndrome. J. Intellect. Disabil. Res. 44, 654–665. 10.1111/j.1365-2788.2000.00305.x 11115020

[B136] Kumar-Singh S. De Jonghe C. Cruts M. Kleinert R. Wang R. Mercken M.. (2000). Nonfibrillar diffuse amyloid deposition due to a gamma(42)-secretase site mutation points to an essential role for N-truncated A beta(42) in Alzheimer's disease. Hum. Mol. Genet. 9, 2589–2598. 10.1093/hmg/9.18.2589 11063718

[B137] LaFerla F. M. Green K. N. Oddo S. (2007). Intracellular amyloid-beta in Alzheimer's disease. Nat. Rev. Neurosci. 8, 499–509. 10.1038/nrn2168 17551515

[B138] Landt J. D'Abrera J. C. Holland A. J. Aigbirhio F. I. Fryer T. D. Canales R.. (2011). Using positron emission tomography and Carbon 11-labeled Pittsburgh Compound B to image brain Fibrillar β-amyloid in adults with down syndrome: safety, acceptability, and feasibility. Arch. Neurol. 68, 890–896. 10.1001/archneurol.2011.36 21403005

[B139] Lassalle J. M. Halley H. Daumas S. Verret L. Francés B. (2008). Effects of the genetic background on cognitive performances of TG2576 mice. Behav. Brain Res. 191, 104–110. 10.1016/j.bbr.2008.03.017 18433892

[B140] Lee L. Dale E. Staniszewski A. Zhang H. Saeed F. Sakurai M.. (2014). Regulation of synaptic plasticity and cognition by SUMO in normal physiology and Alzheimer's disease. Sci. Rep. 4:7190. 10.1038/srep07190 25448527 PMC4250909

[B141] Lehman E. J. H. Kulnane L. S. Gao Y. Petriello M. C. Pimpis K. M. Younkin L.. (2003). Genetic background regulates beta-amyloid precursor protein processing and beta-amyloid deposition in the mouse. Hum. Mol. Genet. 12, 2949–2956. 10.1093/hmg/ddg322 14506131

[B142] Lesné S. Koh M. T. Kotilinek L. Kayed R. Glabe C. G. Yang A.. (2006). A specific amyloid-beta protein assembly in the brain impairs memory. Nature 440, 352–357. 10.1038/nature04533 16541076

[B143] Letourneau A. Santoni F. A. Bonilla X. Sailani M. R. Gonzalez D. Kind J.. (2014). Domains of genome-wide gene expression dysregulation in Down's syndrome. Nature 508, 345–350. 10.1038/nature13200 24740065

[B144] Leverenz J. B. Raskind M. A. (1998). Early amyloid deposition in the medial temporal lobe of young Down syndrome patients: a regional quantitative analysis. Exp. Neurol. 150, 296–304. 10.1006/exnr.1997.6777 9527899

[B145] Li Z. Yu T. Morishima M. Pao A. LaDuca J. Conroy J.. (2007). Duplication of the entire 22.9 Mb human chromosome 21 syntenic region on mouse chromosome 16 causes cardiovascular and gastrointestinal abnormalities. Hum. Mol. Genet. 16, 1359–1366. 10.1093/hmg/ddm086 17412756

[B146] Liu C. Belichenko P. V. Zhang L. Fu D. Kleschevnikov A. M. Baldini A.. (2011). Mouse models for Down syndrome-associated developmental cognitive disabilities. Dev. Neurosci. 33, 404–413. 10.1159/000329422 21865664 PMC3254039

[B147] Liu C. Morishima M. Jiang X. Yu T. Meng K. Ray D.. (2014). Engineered chromosome-based genetic mapping establishes a 3.7 Mb critical genomic region for Down syndrome-associated heart defects in mice. Hum. Genet. 133, 743–753. 10.1007/s00439-013-1407-z 24362460 PMC4024075

[B148] Liu F. Liang Z. Wegiel J. Hwang Y.-W. Iqbal K. Grundke-Iqbal I.. (2008). Overexpression of Dyrk1A contributes to neurofibrillary degeneration in Down syndrome. FASEB J. 22, 3224–3233. 10.1096/fj.07-104539 18509201 PMC2518253

[B149] Loane M. Morris J. K. Addor M.-C. Arriola L. Budd J. Doray B.. (2013). Twenty-year trends in the prevalence of Down syndrome and other trisomies in Europe: impact of maternal age and prenatal screening. Eur. J. Hum. Genet. 21, 27–33. 10.1038/ejhg.2012.94 22713804 PMC3522199

[B150] Lockrow J. Boger H. Bimonte-Nelson H. Granholm A. C. (2011a). Effects of long-term memantine on memory and neuropathology in Ts65Dn mice, a model for Down syndrome. Behav. Brain Res. 221, 610–622. 10.1016/j.bbr.2010.03.036 20363261 PMC2928411

[B151] Lockrow J. Boger H. Gerhardt G. Aston-Jones G. Bachman D. Granholm A. C. (2011b). A noradrenergic lesion exacerbates neurodegeneration in a down syndrome mouse model. J. Alzheimers Dis. 23, 471–489. 10.3233/JAD-2010-101218 21098982 PMC3991557

[B152] Lockrow J. Prakasam A. Huang P. Bimonte-Nelson H. Sambamurti K. Granholm A. C. (2009). Cholinergic degeneration and memory loss delayed by vitamin E in a Down syndrome mouse model. Exp. Neurol. 216, 278–289. 10.1016/j.expneurol.2008.11.021 19135442 PMC2704550

[B153] Lott I. T. (2012). Neurological phenotypes for Down syndrome across the life span. Prog. Brain Res. 197, 101–121. 10.1016/B978-0-444-54299-1.00006-6 22541290 PMC3417824

[B154] Lu J. Esposito G. Scuderi C. Steardo L. Delli-Bovi L. C. Hecht J. L.. (2011). S100B and APP promote a gliocentric shift and impaired neurogenesis in down syndrome neural progenitors. PLoS ONE 6:e22126. 10.1371/journal.pone.0022126 21779383 PMC3133657

[B155] Lu J. Lian G. Zhou H. Esposito G. Steardo L. Delli-Bovi L. C.. (2012). OLIG2 over-expression impairs proliferation of human Down syndrome neural progenitors. Hum. Mol. Genet. 21, 2330–2340. 10.1093/hmg/dds052 22343408 PMC3335315

[B156] Lu T. Aron L. Zullo J. Pan Y. Kim H. Chen Y.. (2014). REST and stress resistance in ageing and Alzheimer disease. Nature 507, 448–454. 10.1038/nature13163 24670762 PMC4110979

[B157] Madeo J. (2013). The Role of Oxidative Stress in Alzheimer's Disease. J. Alzheimers Dis. Park. 03:116 10.4172/2161-0460.1000116

[B158] Maia L. F. Kaeser S. A. Reichwald J. Lambert M. Obermüller U. Schelle J.. (2015). Increased CSF Aβ during the very early phase of cerebral Aβ deposition in mouse models. EMBO Mol. Med. 7, 895–903. 10.15252/emmm.201505026 25978969 PMC4520655

[B159] Mann D. M. Esiri M. M. (1989). The pattern of acquisition of plaques and tangles in the brains of patients under 50 years of age with Down's syndrome. J. Neurol. Sci. 89, 169–179. 2522541 10.1016/0022-510x(89)90019-1

[B160] Mann D. M. (1988a). The pathological association between Down syndrome and Alzheimer disease. Mech. Ageing Dev. 43, 99–136. 10.1016/0047-6374(88)90041-3 2969441

[B161] Mann D. M. (1988b). Alzheimer's disease and Down's syndrome. Histopathology 13, 125–137. 10.1111/j.1365-2559.1988.tb02018.x 2971602

[B162] Margallo-Lana M. L. Moore P. B. Kay D. W. K. Perry R. H. Reid B. E. Berney T. P.. (2007). Fifteen-year follow-up of 92 hospitalized adults with Down's syndrome: incidence of cognitive decline, its relationship to age and neuropathology. J. Intellect. Disabil. Res. 51, 463–477. 10.1111/j.1365-2788.2006.00902.x 17493029

[B163] Martin K. R. Corlett A. Dubach D. Mustafa T. Coleman H. A. Parkington H. C.. (2012). Over-expression of RCAN1 causes Down syndrome-like hippocampal deficits that alter learning and memory. Hum. Mol. Genet. 21, 3025–3041. 10.1093/hmg/dds134 22511596

[B164] Martin S. B. Dowling A. L. Lianekhammy J. Lott I. T. Doran E. Murphy M. P.. (2014). Synaptophysin and Synaptojanin-1 in Down Syndrome are Differentially Affected by Alzheimer's Disease. J. Alzheimers Dis. 42, 767–775. 10.3233/jad-140795 24927707 PMC4392817

[B165] Martínez-Cué C. Delatour B. Potier M.-C. (2014). Treating enhanced GABAergic inhibition in Down syndrome: use of GABA α5-selective inverse agonists. Neurosci. Biobehav. Rev. 46, 218–227. 10.1016/j.neubiorev.2013.12.008 24412222

[B166] McCarron M. McCallion P. Reilly E. Mulryan N. (2014). A prospective 14-year longitudinal follow-up of dementia in persons with Down syndrome. J. Intellect. Disabil. Res. 58, 61–70. 10.1111/jir.12074 23902161

[B167] McCarron M. O. Nicoll J. A. Graham D. I. (1998). A quartet of Down's syndrome, Alzheimer's disease, cerebral amyloid angiopathy, and cerebral haemorrhage: interacting genetic risk factors. J. Neurol. Neurosurg. Psychiatry 65, 405–406. 10.1136/jnnp.65.3.405 9728967 PMC2170259

[B168] McGeer E. G. Norman M. Boyes B. O'Kusky J. Suzuki J. McGeer P. L. (1985). Acetylcholine and aromatic amine systems in postmortem brain of an infant with Down's syndrome. Exp. Neurol. 87, 557–570. 10.1016/0014-4886(85)90184-0 2857653

[B169] McGeer P. L. Itagaki S. Tago H. McGeer E. G. (1987). Reactive microglia in patients with senile dementia of the Alzheimer type are positive for the histocompatibility glycoprotein HLA-DR. Neurosci. Lett. 79, 195–200. 10.1016/0304-3940(87)90696-3 3670729

[B170] Mendez M. Lim G. (2003). Seizures in elderly patients with dementia: epidemiology and management. Drugs Aging 20, 791–803. 10.2165/00002512-200320110-00001 12964886

[B171] Meraz-Ríos M. A. Franco-Bocanegra D. Toral Rios D. Campos-Peña V. (2014). Early onset Alzheimer's disease and oxidative stress. Oxid. Med. Cell. Longev. 2014, 375968. 10.1155/2014/375968 24669286 PMC3942075

[B172] Mesulam M. Shaw P. Mash D. Weintraub S. (2004). Cholinergic nucleus basalis tauopathy emerges early in the aging-MCI-AD continuum. Ann. Neurol. 55, 815–828. 10.1002/ana.20100 15174015

[B173] Moechars D. Dewachter I. Lorent K. Reversé D. Baekelandt V. Naidu A.. (1999). Early phenotypic changes in transgenic mice that overexpress different mutants of amyloid precursor protein in brain. J. Biol. Chem. 274, 6483–6492. 10037741 10.1074/jbc.274.10.6483

[B174] Mok K. Y. Jones E. L. Hanney M. Harold D. Sims R. Williams J.. (2014). Polymorphisms in BACE2 may affect the age of onset Alzheimer's dementia in Down syndrome. Neurobiol. Aging 35, e1–e5. 10.1016/j.neurobiolaging.2013.12.022 24462566 PMC3969241

[B175] Moore S. Evans L. D. B. Andersson T. Portelius E. Smith J. Dias T. B.. (2015). APP Metabolism Regulates Tau Proteostasis in Human Cerebral Cortex Neurons. Cell Rep. 11, 689–696. 10.1016/j.celrep.2015.03.068 25921538 PMC4431668

[B176] Mori C. Spooner E. T. Wisniewsk K. E. Wisniewski T. M. Yamaguch H. Saido T. C.. (2002). Intraneuronal Abeta42 accumulation in Down syndrome brain. Amyloid 9, 88–102. 12440481

[B177] Mori T. Koyama N. Arendash G. W. Horikoshi-Sakuraba Y. Tan J. Town T. (2010). Overexpression of human S100B exacerbates cerebral amyloidosis and gliosis in the Tg2576 mouse model of Alzheimer's disease. Glia 58, 300–314. 10.1002/glia.20924 19705461 PMC2795105

[B178] Mucke L. Selkoe D. J. (2012). Neurotoxicity of amyloid β-protein: synaptic and network dysfunction. Cold Spring Harb. Perspect. Med. 2, a006338. 10.1101/cshperspect.a006338 22762015 PMC3385944

[B179] Murray A. Letourneau A. Canzonetta C. Stathaki E. Gimelli S. Sloan-Bena F.. (2015). Brief report: isogenic induced pluripotent stem cell lines from an adult with mosaic down syndrome model accelerated neuronal ageing and neurodegeneration. Stem Cells 33, 2077–2084. 10.1002/stem.1968 25694335 PMC4737213

[B180] Murrell J. R. Hake A. M. Quaid K. A. Farlow M. R. Ghetti B. (2000). Early-onset Alzheimer disease caused by a new mutation (V717L) in the amyloid precursor protein gene. Arch. Neurol. 57, 885–887. 10.1001/archneur.57.6.885 10867787

[B181] Musicco M. (2009). Gender differences in the occurrence of Alzheimer's disease. Funct. Neurol. 24, 89–92. 19775536

[B182] Myllykangas L. Wavrant-De Vrieze F. Polvikoski T. Notkola I. L. Sulkava R. Niinisto L.. (2005). Chromosome 21 BACE2 haplotype associates with Alzheimer's disease: a two-stage study. J. Neurol. Sci. 236, 17–24. 10.1016/j.jns.2005.04.008 16023140

[B183] Naito K.-S. Sekijima Y. Ikeda S.-I. (2008). Cerebral amyloid angiopathy-related hemorrhage in a middle-aged patient with Down's syndrome. Amyloid 15, 275–277. 10.1080/13506120802524981 19065301

[B184] Narayan P. J. Lill C. Faull R. Curtis M. A. Dragunow M. (2015). Increased acetyl and total histone levels in post-mortem Alzheimer's disease brain. Neurobiol. Dis. 74, 281–294. 10.1016/j.nbd.2014.11.023 25484284

[B185] Nelson L. D. Orme D. Osann K. Lott I. T. (2001). Neurological changes and emotional functioning in adults with Down Syndrome. J. Intellect. Disabil. Res. 45, 450–456. 10.1046/j.1365-2788.2001.00379.x 11679050

[B186] Ness S. Rafii M. Aisen P. Krams M. Silverman W. Manji H. (2012). Down's syndrome and Alzheimer's disease: towards secondary prevention. Nat. Rev. Drug Discov. 11, 655–656. 10.1038/nrd3822 22935789

[B187] O'Doherty A. Ruf S. Mulligan C. Hildreth V. Errington M. L. Cooke S.. (2005). An aneuploid mouse strain carrying human chromosome 21 with Down syndrome phenotypes. Science 309, 2033–2037. 10.1126/science.1114535 16179473 PMC1378183

[B188] Olson L. E. Richtsmeier J. T. Leszl J. Reeves R. H. (2004). A chromosome 21 critical region does not cause specific Down syndrome phenotypes. Science 306, 687–690. 10.1126/science.1098992 15499018 PMC4019810

[B189] Palop J. J. (2009). Epilepsy and Cognitive Impairments in Alzheimer Disease. Arch. Neurol. 66, 435. 10.1001/archneurol.2009.15 19204149 PMC2812914

[B190] Park J. Yang E. J. Yoon J. H. Chung K. C. (2007). Dyrk1A overexpression in immortalized hippocampal cells produces the neuropathological features of Down syndrome. Mol. Cell Neurosci. 36, 270–279. 10.1016/j.mcn.2007.07.007 17720532

[B191] Parker S. E. Mai C. T. Canfield M. A. Rickard R. Wang Y. Meyer R. E.. (2010). Updated National Birth Prevalence estimates for selected birth defects in the United States, 2004-2006. Birth Defects Res. A. Clin. Mol. Teratol. 88, 1008–1016. 10.1002/bdra.20735 20878909

[B192] Parkinson P. F. Kannangara T. S. Eadie B. D. Burgess B. L. Wellington C. L. Christie B. R. (2009). Cognition, learning behaviour and hippocampal synaptic plasticity are not disrupted in mice over-expressing the cholesterol transporter ABCG1. Lipids Heal. Dis. 8:5 10.1186/1476-511x-8-5 PMC2654451 19239689

[B193] Pasalar P. Najmabadi H. Noorian A. R. Moghimi B. Jannati A. Soltanzadeh A.. (2002). An Iranian family with Alzheimer's disease caused by a novel APP mutation (Thr714Ala). Neurology 58, 1574–1575. 10.1212/WNL.58.10.1574 12034808

[B194] Peacock M. L. Warren J. T. Roses A. D. Fink J. K. (1993). Novel polymorphism in the A4 region of the amyloid precursor protein gene in a patient without Alzheimer's disease. Neurology 43, 1254–1256. 10.1212/WNL.43.6.1254 8170579

[B195] Peng S. Garzon D. J. Marchese M. Klein W. Ginsberg S. D. Francis B. M.. (2009). Decreased brain-derived neurotrophic factor depends on amyloid aggregation state in transgenic mouse models of Alzheimer's disease. J. Neurosci. 29, 9321–9329. 10.1523/JNEUROSCI.4736-08.2009 19625522 PMC3411546

[B196] Pereira P. L. Magnol L. Sahún I. Brault V. Duchon A. Prandini P.. (2009). A new mouse model for the trisomy of the Abcg1-U2af1 region reveals the complexity of the combinatorial genetic code of down syndrome. Hum. Mol. Genet. 18, 4756–4769. 10.1093/hmg/ddp438 19783846 PMC2778371

[B197] Perez-Cruz C. Nolte M. W. van Gaalen M. M. Rustay N. R. Termont A. Tanghe A.. (2011). Reduced spine density in specific regions of CA1 pyramidal neurons in two transgenic mouse models of Alzheimer's disease. J. Neurosci. 31, 3926–3934. 10.1523/JNEUROSCI.6142-10.2011 21389247 PMC6622797

[B198] Pike C. J. Cummings B. J. Cotman C. W. (1995). Early association of reactive astrocytes with senile plaques in Alzheimer's disease. Exp. Neurol. 132, 172–179. 10.1016/0014-4886(95)90022-5 7789457

[B199] Pilotto A. Padovani A. Borroni B. (2013). Clinical, biological, and imaging features of monogenic Alzheimer's Disease. Biomed. Res. Int. 2013:689591. 10.1155/2013/689591 24377094 PMC3860086

[B200] Pollonini G. Gao V. Rabe A. Palminiello S. Albertini G. Alberini C. M. (2008). Abnormal expression of synaptic proteins and neurotrophin-3 in the Down syndrome mouse model Ts65Dn. Neuroscience 156, 99–106. 10.1016/j.neuroscience.2008.07.025 18703118 PMC2613315

[B201] Portelius E. Hölttä M. Soininen H. Bjerke M. Zetterberg H. Westerlund A.. (2014a). Altered cerebrospinal fluid levels of amyloid β and amyloid precursor-like protein 1 peptides in Down's syndrome. NeuroMol. Med. 16, 510–516. 10.1007/s12017-014-8302-1 24740518

[B202] Portelius E. Soininen H. Andreasson U. Zetterberg H. Persson R. Karlsson G.. (2014b). Exploring Alzheimer molecular pathology in Down's syndrome cerebrospinal fluid. Neurodegener. Dis. 14, 98–106. 10.1159/000358800 24992945

[B203] Powell D. Caban-Holt A. Jicha G. Robertson W. Davis R. Gold B. T.. (2014). Frontal white matter integrity in adults with Down syndrome with and without dementia. Neurobiol. Aging 35, 1562–1569. 10.1016/j.neurobiolaging.2014.01.137 24582640 PMC3992921

[B204] Pozueta J. Lefort R. Shelanski M. L. (2013). Synaptic changes in Alzheimer's disease and its models. Neuroscience 251, 51–65. 10.1016/j.neuroscience.2012.05.050 22687952

[B205] Prasher V. P. Farrer M. J. Kessling A. M. Fisher E. M. West R. J. Barber P. C.. (1998). Molecular mapping of Alzheimer-type dementia in Down's syndrome. Ann. Neurol. 43, 380–383. 10.1002/ana.410430316 9506555

[B206] Prasher V. P. Krishnan V. H. R. (1993). Age of onset and duration of dementia in people with down syndrome: integration of 98 reported cases in the literature. Int. J. Geriatr. Psychiatry 8, 915–922. 10.1002/gps.930081105

[B207] Pucharcos C. Fuentes J. J. Casas C. de la Luna S. Alcantara S. Arbones M. L.. (1999). Alu-splice cloning of human Intersectin (ITSN), a putative multivalent binding protein expressed in proliferating and differentiating neurons and overexpressed in Down syndrome. Eur. J. Hum. Genet. 7, 704–712. 10.1038/sj.ejhg.5200356 10482960

[B208] Querfurth H. W. LaFerla F. M. (2010). Alzheimer's disease. N. Engl. J. Med. 362, 329–344. 10.1056/NEJMra0909142 20107219

[B209] Reeves R. H. Irving N. G. Moran T. H. Wohn A. Kitt C. Sisodia S. S.. (1995). A mouse model for Down syndrome exhibits learning and behaviour deficits. Nat. Genet. 11, 177–184. 10.1038/ng1095-177 7550346

[B210] Reinholdt L. G. Ding Y. Gilbert G. J. Gilbert G. T. Czechanski A. Solzak J. P.. (2011). Molecular characterization of the translocation breakpoints in the Down syndrome mouse model Ts65Dn. Mamm. Genome 22, 685–691. 10.1007/s00335-011-9357-z 21953412 PMC3505986

[B211] Reynolds G. P. Warner C. E. J. (1988). Amino acid neurotransmitter deficits in adult Down's syndrome brain tissue. Neurosci. Lett. 94, 224–227. 10.1016/0304-3940(88)90299-6 2907377

[B212] Rodríguez-Arellano J. J. Parpura V. Zorec R. Verkhratsky A. (2015). Astrocytes in physiological aging and Alzheimer's disease. Neuroscience. [Epub ahead of print]. 10.1016/j.neuroscience.2015.01.007 25595973

[B213] Rovelet-Lecrux A. Frebourg T. Tuominen H. Majamaa K. Campion D. Remes A. M. (2007). APP locus duplication in a Finnish family with dementia and intracerebral haemorrhage. J. Neurol. Neurosurg. Psychiatry 78, 1158–1159. 10.1136/jnnp.2006.113514 17442758 PMC2117532

[B214] Rovelet-Lecrux A. Hannequin D. Raux G. Le Meur N. Laquerrière A. Vital A.. (2006). APP locus duplication causes autosomal dominant early-onset Alzheimer disease with cerebral amyloid angiopathy. Nat. Genet. 38, 24–26. 10.1038/ng1718 16369530

[B215] Royston M. C. McKenzie J. E. Gentleman S. M. Sheng J. G. Mann D. M. Griffin W. S.. (1999). Overexpression of S100beta in Down's syndrome: correlation with patient age and with beta-amyloid deposition. Neuropathol. Appl. Neurobiol. 25, 387–393. 10.1046/j.1365-2990.1999.00196.x 10564528

[B216] Ruparelia A. Pearn M. L. Mobley W. C. (2013). Aging and intellectual disability: insights from mouse models of Down syndrome. Dev. Disabil. Res. Rev. 18, 43–50. 10.1002/ddrr.1127 23949828

[B217] Rustay N. R. Cronin E. A. Curzon P. Markosyan S. Bitner R. S. Ellis T. A.. (2010). Mice expressing the Swedish APP mutation on a 129 genetic background demonstrate consistent behavioral deficits and pathological markers of Alzheimer's disease. Brain Res. 1311, 136–147. 10.1016/j.brainres.2009.11.040 19944081

[B218] Ryan N. S. Rossor M. N. (2010). Correlating familial Alzheimer's disease gene mutations with clinical phenotype. Biomark. Med. 4, 99–112. 10.2217/bmm.09.92 20387306 PMC3937872

[B219] Ryoo S. R. Cho H. J. Lee H. W. Jeong H. K. Radnaabazar C. Kim Y. S.. (2008). Dual-specificity tyrosine(Y)-phosphorylation regulated kinase 1A-mediated phosphorylation of amyloid precursor protein: evidence for a functional link between Down syndrome and Alzheimer's disease. J. Neurochem. 104, 1333–1344. 10.1111/j.1471-4159.2007.05075.x 18005339

[B220] Ryoo S. R. Jeong H. K. Radnaabazar C. Yoo J. J. Cho H. J. Lee H. W.. (2007). DYRK1A-mediated hyperphosphorylation of Tau. A functional link between Down syndrome and Alzheimer disease. J. Biol. Chem. 282, 34850–34857. 10.1074/jbc.M707358200 17906291

[B221] Ryu Y. S. Park S. Y. Jung M. S. Yoon S. H. Kwen M. Y. Lee S. Y.. (2010). Dyrk1A-mediated phosphorylation of Presenilin 1: a functional link between Down syndrome and Alzheimer's disease. J. Neurochem. 115, 574–584. 10.1111/j.1471-4159.2010.06769.x 20456003

[B222] Sabbagh M. N. Chen K. Rogers J. Fleisher A. S. Liebsack C. Bandy D.. (2015). Florbetapir PET, FDG PET, and MRI in Down syndrome individuals with and without Alzheimer's dementia. Alzheimers Dementia 11, 994–1004. 10.1016/j.jalz.2015.01.006 25849033 PMC4543530

[B223] Sago H. Carlson E. J. Smith D. J. Kilbridge J. Rubin E. M. Mobley W. C.. (1998). Ts1Cje, a partial trisomy 16 mouse model for Down syndrome, exhibits learning and behavioral abnormalities. Proc. Natl. Acad. Sci. U.S.A. 95, 6256–6261. 10.1073/pnas.95.11.6256 9600952 PMC27649

[B224] Saito T. Matsuba Y. Mihira N. Takano J. Nilsson P. Itohara S.. (2014). Single App knock-in mouse models of Alzheimer's disease. Nat. Neurosci. 17, 661–663. 10.1038/nn.3697 24728269

[B225] Saito Y. Oka A. Mizuguchi M. Motonaga K. Mori Y. Becker L. E.. (2000). The developmental and aging changes of Down's syndrome cell adhesion molecule expression in normal and Down's syndrome brains. Acta Neuropathol. 100, 654–664. 10.1007/s004010000230 11078217

[B226] Salehi A. Delcroix J.-D. Belichenko P. V. Zhan K. Wu C. Valletta J. S.. (2006). Increased App expression in a mouse model of Down's syndrome disrupts NGF transport and causes cholinergic neuron degeneration. Neuron 51, 29–42. 10.1016/j.neuron.2006.05.022 16815330

[B227] Sanchez M. M. Moghadam S. Naik P. Martin K. J. Salehi A. (2011). Hippocampal network alterations in Alzheimer's disease and Down syndrome: from structure to therapy. J. Alzheimers Dis. 26(Suppl 3), 29–47. 10.3233/JAD-2011-0050 21971449

[B228] Sanchez P. E. Zhu L. Verret L. Vossel K. A. Orr A. G. Cirrito J. R.. (2012). Levetiracetam suppresses neuronal network dysfunction and reverses synaptic and cognitive deficits in an Alzheimer's disease model. Proc. Natl. Acad. Sci. U.S.A. 109, E2895–E2903. 10.1073/pnas.1121081109 22869752 PMC3479491

[B229] Sanders N. C. Williams D. K. Wenger G. R. (2009). Does the learning deficit observed under an incremental repeated acquisition schedule of reinforcement in Ts65Dn mice, a model for Down syndrome, change as they age? Behav. Brain Res. 203, 137–142. 10.1016/j.bbr.2009.04.031 19409933 PMC2700176

[B230] Sangani M. Shahid A. Amina S. Koubeissi M. (2010). Improvement of myoclonic epilepsy in Down syndrome treated with levetiracetam. Epileptic Disord. 12, 151–154. 10.1684/epd.2010.0306 20483713

[B231] Scheff S. W. Price D. A. Schmitt F. A. DeKosky S. T. Mufson E. J. (2007). Synaptic alterations in CA1 in mild Alzheimer disease and mild cognitive impairment. Neurology 68, 1501–1508. 10.1212/01.wnl.0000260698.46517.8f 17470753

[B232] Schupf N. Lee A. Park N. Dang L.-H. Pang D. Yale A.. (2015). Candidate genes for Alzheimer's disease are associated with individual differences in plasma levels of beta amyloid peptides in adults with Down syndrome. Neurobiol. Aging 36, 2907.e1–2907.e10. 10.1016/j.neurobiolaging.2015.06.020 26166206 PMC4562880

[B233] Seidl R. Cairns N. Singewald N. Kaehler S. T. Lubec G. (2001). Differences between GABA levels in Alzheimer's disease and Down syndrome with Alzheimer-like neuropathology. Naunyn. Schmiedebergs. Arch. Pharmacol. 363, 139–145. 10.1007/s002100000346 11218066

[B234] Seo H. Isacson O. (2005). Abnormal APP, cholinergic and cognitive function in Ts65Dn Down's model mice. Exp. Neurol. 193, 469–480. 10.1016/j.expneurol.2004.11.017 15869949

[B235] Serrano-Pozo A. Frosch M. P. Masliah E. Hyman B. T. (2011). Neuropathological alterations in Alzheimer disease. Cold Spring Harb. Perspect. Med. 1:a006189. 10.1101/cshperspect.a006189 22229116 PMC3234452

[B236] Shapiro L. A. Marks A. Whitaker-Azmitia P. M. (2004). Increased clusterin expression in old but not young adult S100B transgenic mice: evidence of neuropathological aging in a model of Down Syndrome. Brain Res. 1010, 17–21. 10.1016/j.brainres.2003.12.057 15126113

[B237] Shapiro L. A. Whitaker-Azmitia P. M. (2004). Expression levels of cytoskeletal proteins indicate pathological aging of S100B transgenic mice: an immunohistochemical study of MAP-2, drebrin and GAP-43. Brain Res. 1019, 39–46. 10.1016/j.brainres.2004.05.100 15306236

[B238] Sheehan R. Sinai A. Bass N. Blatchford P. Bohnen I. Bonell S.. (2015). Dementia diagnostic criteria in Down syndrome. Int. J. Geriatr. Psychiatry 30, 857–863. 10.1002/gps.4228 25363568 PMC4678599

[B239] Sheng J. G. Mrak R. E. Griffin W. S. (1994). S100 beta protein expression in Alzheimer disease: potential role in the pathogenesis of neuritic plaques. J. Neurosci. Res. 39, 398–404. 10.1002/jnr.490390406 7884819

[B240] Sheppard O. Plattner F. Rubin A. Slender A. Linehan J. M. Brandner S.. (2012). Altered regulation of tau phosphorylation in a mouse model of down syndrome aging. Neurobiol. Aging 33, e31–e44. 10.1016/j.neurobiolaging.2011.06.025 21843906 PMC3314962

[B241] Shi J. Zhang T. Zhou C. Chohan M. O. Gu X. Wegiel J.. (2008). Increased dosage of Dyrk1A alters alternative splicing factor (ASF)-regulated alternative splicing of tau in Down syndrome. J. Biol. Chem. 283, 28660–28669. 10.1074/jbc.M802645200 18658135 PMC2568927

[B242] Shi Y. Kirwan P. Smith J. MacLean G. Orkin S. H. Livesey F. J. (2012). A human stem cell model of early Alzheimer's disease pathology in Down syndrome. Sci. Transl. Med. 4, 124ra29. 10.1126/scitranslmed.3003771 22344463 PMC4129935

[B243] Shichiri M. Yoshida Y. Ishida N. Hagihara Y. Iwahashi H. Tamai H.. (2011). α-Tocopherol suppresses lipid peroxidation and behavioral and cognitive impairments in the Ts65Dn mouse model of Down syndrome. Free Radic. Biol. Med. 50, 1801–1811. 10.1016/j.freeradbiomed.2011.03.023 21447382

[B244] Shukkur E. A. Shimohata A. Akagi T. Yu W. Yamaguchi M. Murayama M.. (2006). Mitochondrial dysfunction and tau hyperphosphorylation in Ts1Cje, a mouse model for Down syndrome. Hum. Mol. Genet. 15, 2752–2762. 10.1093/hmg/ddl211 16891409

[B245] Siarey R. J. Kline-Burgess A. Cho M. Balbo A. Best T. K. Harashima C.. (2006). Altered signaling pathways underlying abnormal hippocampal synaptic plasticity in the Ts65Dn mouse model of Down syndrome. J. Neurochem. 98, 1266–1277. 10.1111/j.1471-4159.2006.03971.x 16895585

[B246] Sims R. Hollingworth P. Moskvina V. Dowzell K. O'Donovan M. C. Powell J.. (2009). Evidence that variation in the oligodendrocyte lineage transcription factor 2 (OLIG2) gene is associated with psychosis in Alzheimer's disease. Neurosci. Lett. 461, 54–59. 10.1016/j.neulet.2009.05.051 19477230

[B247] Sleegers K. Brouwers N. Gijselinck I. Theuns J. Goossens D. Wauters J.. (2006). APP duplication is sufficient to cause early onset Alzheimer's dementia with cerebral amyloid angiopathy. Brain 129, 2977–2983. 10.1093/brain/awl203 16921174

[B248] Stargardt A. Swaab D. F. Bossers K. (2015). The storm before the quiet: neuronal hyperactivity and Aβ in the presymptomatic stages of Alzheimer's disease. Neurobiol. Aging 36, 1–11. 10.1016/j.neurobiolaging.2014.08.014 25444609

[B249] Streit W. J. Braak H. Xue Q.-S. Bechmann I. (2009). Dystrophic (senescent) rather than activated microglial cells are associated with tau pathology and likely precede neurodegeneration in Alzheimer's disease. Acta Neuropathol. 118, 475–485. 10.1007/s00401-009-0556-6 19513731 PMC2737117

[B250] Sturchler-Pierrat C. Abramowski D. Duke M. Wiederhold K. H. Mistl C. Rothacher S.. (1997). Two amyloid precursor protein transgenic mouse models with Alzheimer disease-like pathology. Proc. Natl. Acad. Sci. U.S.A. 94, 13287–13292. 10.1073/pnas.94.24.13287 9371838 PMC24301

[B251] Sun X. He G. Song W. (2006). BACE2, as a novel APP theta-secretase, is not responsible for the pathogenesis of Alzheimer's disease in Down syndrome. FASEB J. 20, 1369–1376. 10.1096/fj.05-5632com 16816112

[B252] Sun X. Wu Y. Chen B. Zhang Z. Zhou W. Tong Y.. (2011). Regulator of calcineurin 1 (RCAN1) facilitates neuronal apoptosis through caspase-3 activation. J. Biol. Chem. 286, 9049–9062. 10.1074/jbc.M110.177519 21216952 PMC3059004

[B253] Sun X. Wu Y. Herculano B. Song W. (2014). RCAN1 Overexpression exacerbates calcium overloading-induced neuronal apoptosis. PLoS ONE 9:e95471. 10.1371/journal.pone.0095471 24751678 PMC3994074

[B254] Szot P. Van Dam D. White S. S. Franklin A. Staufenbiel M. De Deyn P. P. (2009). Age-dependent changes in noradrenergic locus coeruleus system in wild-type and APP23 transgenic mice. Neurosci. Lett. 463, 93–97. 10.1016/j.neulet.2009.07.055 19631722

[B255] Takahashi R. H. Milner T. A. Li F. Nam E. E. Edgar M. A. Yamaguchi H.. (2002). Intraneuronal Alzheimer abeta42 accumulates in multivesicular bodies and is associated with synaptic pathology. Am. J. Pathol. 161, 1869–1879. 10.1016/S0002-9440(10)64463-X 12414533 PMC1850783

[B256] Takano T. Han X. Deane R. Zlokovic B. Nedergaard M. (2007). Two-photon imaging of astrocytic Ca2+ signaling and the microvasculature in experimental mice models of Alzheimer's disease. Ann. N. Y. Acad. Sci. 1097, 40–50. 10.1196/annals.1379.004 17413008

[B257] Tansley G. H. Burgess B. L. Bryan M. T. Su Y. Hirsch-Reinshagen V. Pearce J.. (2007). The cholesterol transporter ABCG1 modulates the subcellular distribution and proteolytic processing of beta-amyloid precursor protein. J. Lipid Res. 48, 1022–1034. 10.1194/jlr.M600542-JLR200 17293612

[B258] Tanzi R. E. (2012). The genetics of Alzheimer disease. Cold Spring Harb. Perspect. Med. 2:a006296. 10.1101/cshperspect.a006296 23028126 PMC3475404

[B259] Thal D. R. Rüb U. Orantes M. Braak H. (2002). Phases of A β-deposition in the human brain and its relevance for the development of AD. Neurology 58, 1791–1800. 10.1212/WNL.58.12.1791 12084879

[B260] Tybulewicz V. L. J. Fisher E. M. C. (2006). New techniques to understand chromosome dosage: mouse models of aneuploidy. Hum. Mol. Genet. 15, R103–R109. 10.1093/hmg/ddl179 16987872

[B261] Tyrrell J. Cosgrave M. McCarron M. McPherson J. Calvert J. Kelly A.. (2001). Dementia in people with Down's syndrome. Int. J. Geriatr. Psychiatry 16, 1168–1174. 10.1002/gps.502 11748777

[B262] Vacík T. Ort M. Gregorová S. Strnad P. Blatny R. Conte N.. (2005). Segmental trisomy of chromosome 17: a mouse model of human aneuploidy syndromes. Proc. Natl. Acad. Sci. U.S.A. 102, 4500–4505. 10.1073/pnas.0500802102 15755806 PMC552979

[B263] Van Dam D. D'Hooge R. Staufenbiel M. Van Ginneken C. Van Meir F. De Deyn P. P. (2003). Age-dependent cognitive decline in the APP23 model precedes amyloid deposition. Eur. J. Neurosci. 17, 388–396. 10.1046/j.1460-9568.2003.02444.x 12542676

[B264] Van Dam D. Marescau B. Engelborghs S. Cremers T. Mulder J. Staufenbiel M.. (2005). Analysis of cholinergic markers, biogenic amines, and amino acids in the CNS of two APP overexpression mouse models. Neurochem. Int. 46, 409–422. 10.1016/j.neuint.2004.11.005 15737439

[B265] Veerappan C. S. Sleiman S. Coppola G. (2013). Epigenetics of Alzheimer's disease and frontotemporal dementia. Neurotherapeutics 10, 709–721. 10.1007/s13311-013-0219-0 24150812 PMC3805876

[B266] Verret L. Mann E. O. Hang G. B. Barth A. M. I. Cobos I. Ho K.. (2012). Inhibitory interneuron deficit links altered network activity and cognitive dysfunction in Alzheimer model. Cell 149, 708–721. 10.1016/j.cell.2012.02.046 22541439 PMC3375906

[B267] Vilardell M. Rasche A. Thormann A. Maschke-Dutz E. Pérez-Jurado L. A. Lehrach H.. (2011). Meta-analysis of heterogeneous Down Syndrome data reveals consistent genome-wide dosage effects related to neurological processes. BMC Genomics 12:229. 10.1186/1471-2164-12-229 21569303 PMC3110572

[B268] Voronov S. V. Frere S. G. Giovedi S. Pollina E. A. Borel C. Zhang H.. (2008). Synaptojanin 1-linked phosphoinositide dyshomeostasis and cognitive deficits in mouse models of Down's syndrome. Proc. Natl. Acad. Sci. U.S.A. 105, 9415–9420. 10.1073/pnas.0803756105 18591654 PMC2453748

[B269] Vossel K. A. Beagle A. J. Rabinovici G. D. Shu H. Lee S. E. Naasan G.. (2013). Seizures and epileptiform activity in the early stages of Alzheimer disease. JAMA Neurol. 70, 1158–1166. 10.1001/jamaneurol.2013.136 23835471 PMC4013391

[B270] Wallin A. K. Blennow K. Andreasen N. Minthon L. (2006). CSF biomarkers for Alzheimer's Disease: levels of beta-amyloid, tau, phosphorylated tau relate to clinical symptoms and survival. Dement. Geriatr. Cogn. Disord. 21, 131–138. 10.1159/000090631 16391474

[B271] Wang X. Huang T. Zhao Y. Zheng Q. Thompson R. C. Bu G.. (2014). Sorting nexin 27 regulates Abeta production through modulating gamma-secretase activity. Cell Rep. 9, 1023–1033. 10.1016/j.celrep.2014.09.037 25437557 PMC4328673

[B272] Wang X. Zhao Y. Zhang X. Badie H. Zhou Y. Mu Y.. (2013). Loss of sorting nexin 27 contributes to excitatory synaptic dysfunction by modulating glutamate receptor recycling in Down's syndrome. Nat. Med. 19, 473–480. 10.1038/nm.3117 23524343 PMC3911880

[B273] Webster S. J. Bachstetter A. D. Nelson P. T. Schmitt F. A. Van Eldik L. J. (2014). Using mice to model Alzheimer's dementia: an overview of the clinical disease and the preclinical behavioral changes in 10 mouse models. Front. Genet. 5:88. 10.3389/fgene.2014.00088 24795750 PMC4005958

[B274] Wegiel J. Dowjat K. Kaczmarski W. Kuchna I. Nowicki K. Frackowiak J.. (2008). The role of overexpressed DYRK1A protein in the early onset of neurofibrillary degeneration in Down syndrome. Acta Neuropathol. 116, 391–407. 10.1007/s00401-008-0419-6 18696092 PMC2656568

[B275] Wegiel J. Kaczmarski W. Barua M. Kuchna I. Nowicki K. Wang K. C.. (2011). Link between DYRK1A overexpression and several-fold enhancement of neurofibrillary degeneration with 3-repeat tau protein in Down syndrome. J. Neuropathol. Exp. Neurol. 70, 36–50. 10.1097/NEN.0b013e318202bfa1 21157379 PMC3083064

[B276] Weintraub S. Wicklund A. H. Salmon D. P. (2012). The neuropsychological profile of Alzheimer disease. Cold Spring Harb. Perspect. Med. 2, a006171. 10.1101/cshperspect.a006171 22474609 PMC3312395

[B277] Westerman M. A. Cooper-Blacketer D. Mariash A. Kotilinek L. Kawarabayashi T. Younkin L. H.. (2002). The relationship between Abeta and memory in the Tg2576 mouse model of Alzheimer's disease. J. Neurosci. 22, 1858–1867. 11880515 10.1523/JNEUROSCI.22-05-01858.2002 PMC6758862

[B278] Westmark C. J. Westmark P. R. Malter J. S. (2010). Alzheimer's disease and Down syndrome rodent models exhibit audiogenic seizures. J. Alzheimer's Dis. 20, 1009–1013. 10.3233/JAD-2010-100087 20413855 PMC2915889

[B279] Wilmot B. McWeeney S. K. Nixon R. R. Montine T. J. Laut J. Harrington C. A.. (2008). Translational gene mapping of cognitive decline. Neurobiol. Aging 29, 524–541. 10.1016/j.neurobiolaging.2006.11.008 17174450 PMC2684335

[B280] Wiseman F. K. Al-Janabi T. Hardy J. Karmiloff-Smith A. Nizetic D. Tybulewicz V. L. J.. (2015). A genetic cause of Alzheimer disease: mechanistic insights from Down syndrome. Nat. Rev. Neurosci. 16, 564–574. 10.1038/nrn3983 26243569 PMC4678594

[B281] Wisniewski K. E. Wisniewski H. M. Wen G. Y. (1985). Occurrence of neuropathological changes and dementia of Alzheimer's disease in Down's syndrome. Ann. Neurol. 17, 278–282. 10.1002/ana.410170310 3158266

[B282] Witton J. Padmashri R. Zinyuk L. E. Popov V. I. Kraev I. Line S. J.. (2015). Hippocampal circuit dysfunction in the Tc1 mouse model of Down syndrome. Nat. Neurosci. 18, 1291–1298. 10.1038/nn.4072 26237367 PMC4552261

[B283] Wolvetang E. J. Wilson T. J. Sanij E. Busciglio J. Hatzistavrou T. Seth A.. (2003a). ETS2 overexpression in transgenic models and in Down syndrome predisposes to apoptosis via the p53 pathway. Hum. Mol. Genet. 12, 247–255. 10.1093/hmg/ddg015 12554679

[B284] Wolvetang E. W. Bradfield O. M. Tymms M. Zavarsek S. Hatzistavrou T. Kola I.. (2003b). The chromosome 21 transcription factor ETS2 transactivates the beta-APP promoter: implications for Down syndrome. Biochim. Biophys. Acta 1628, 105–110. 10.1016/S0167-4781(03)00121-0 12890557

[B285] Wu J. Morris J. K. (2013). The population prevalence of Down's syndrome in England and Wales in 2011. Eur. J. Hum. Genet. 21, 1016–1019. 10.1038/ejhg.2012.294 23321618 PMC3746270

[B286] Xue Q.-S. Streit W. J. (2011). Microglial pathology in Down syndrome. Acta Neuropathol. 122, 455–466. 10.1007/s00401-011-0864-5 21847625

[B287] Yang Q. Rasmussen S. A. Friedman J. M. (2002). Mortality associated with Down's syndrome in the USA from 1983 to 1997: a population-based study. Lancet 359, 1019–1025. 10.1016/S0140-6736(02)08092-3 11937181

[B288] Ye X. Tai W. Zhang D. (2012). The early events of Alzheimer's disease pathology: from mitochondrial dysfunction to BDNF axonal transport deficits. Neurobiol. Aging 33, e1–e10. 10.1016/j.neurobiolaging.2011.11.004 22212405

[B289] Yu T. Liu C. Belichenko P. Clapcote S. J. Li S. Pao A.. (2010b). Effects of individual segmental trisomies of human chromosome 21 syntenic regions on hippocampal long-term potentiation and cognitive behaviors in mice. Brain Res. 1366, 162–171. 10.1016/j.brainres.2010.09.107 20932954 PMC3027718

[B290] Yu T. Li Z. Jia Z. Clapcote S. J. Liu C. Li S.. (2010a). A mouse model of Down syndrome trisomic for all human chromosome 21 syntenic regions. Hum. Mol. Genet. 19, 2780–2791. 10.1093/hmg/ddq179 20442137 PMC2893810

[B291] Zarow C. Lyness S. A. Mortimer J. A. Chui H. C. (2003). Neuronal Loss Is Greater in the Locus Coeruleus Than Nucleus Basalis and Substantia Nigra in Alzheimer and Parkinson Diseases. Arch. Neurol. 60, 337. 10.1001/archneur.60.3.337 12633144

[B292] Zhang K. Schrag M. Crofton A. Trivedi R. Vinters H. Kirsch W. (2012a). Targeted proteomics for quantification of histone acetylation in Alzheimer's disease. Proteomics 12, 1261–1268. 10.1002/pmic.201200010 22577027 PMC6812507

[B293] Zhang L. Fu D. Belichenko P. V. Liu C. Kleschevnikov A. M. Pao A.. (2012b). Genetic analysis of Down syndrome facilitated by mouse chromosome engineering. Bioeng. Bugs 3, 8–12. 10.4161/bbug.3.1.17696 22126738 PMC3329253

[B294] Zhang Y. Q. Sarge K. D. (2008). Sumoylation of amyloid precursor protein negatively regulates Abeta aggregate levels. Biochem. Biophys. Res. Commun. 374, 673–678. 10.1016/j.bbrc.2008.07.109 18675254 PMC2596940

[B295] Zigman W. B. (2013). Atypical aging in Down syndrome. Dev. Disabil. Res. Rev. 18, 51–67. 10.1002/ddrr.1128 23949829

[B296] Zis P. Dickinson M. Shende S. Walker Z. Strydom A. (2012). Oxidative stress and memory decline in adults with Down syndrome: longitudinal study. J. Alzheimer's Dis. 31, 277–283. 10.3233/JAD-2012-120073 22561328

